# Integration of single-cell RNA sequencing and bulk RNA transcriptome sequencing reveals a heterogeneous immune landscape and pivotal cell subpopulations associated with colorectal cancer prognosis

**DOI:** 10.3389/fimmu.2023.1184167

**Published:** 2023-08-22

**Authors:** Qian Zhang, Yang Liu, Xinyu Wang, Cheng Zhang, Mingxiao Hou, Yunen Liu

**Affiliations:** ^1^College of Medicine and Biological Information Engineering, Northeastern University, Shenyang, Liaoning, China; ^2^Shuren International College, Shenyang Medical College, Shenyang, Liaoning, China; ^3^Department of General Surgery, General Hospital of Northern Theater Command, Shenyang, China; ^4^The Second Affiliated Hospital of Shenyang Medical College, The Veterans General Hospital of Liaoning Province, Shenyang, Liaoning, China

**Keywords:** single cell, immune landscape, colorectal cancer, tumor-associated macrophages, Treg, plasma B cell

## Abstract

**Introduction:**

Colorectal cancer (CRC) is a highly heterogeneous cancer. The molecular and cellular characteristics differ between the colon and rectal cancer type due to the differences in their anatomical location and pathological properties. With the advent of single-cell sequencing, it has become possible to analyze inter- and intra-tumoral tissue heterogeneities.

**Methods:**

A comprehensive CRC immune atlas, comprising 62,398 immune cells, was re-structured into 33 immune cell clusters at the single-cell level. Further, the immune cell lineage heterogeneity of colon, rectal, and paracancerous tissues was explored. Simultaneously, we characterized the TAM phenotypes and analyzed the transcriptomic factor regulatory network of each macrophage subset using SCENIC. In addition, monocle2 was used to elucidate the B cell developmental trajectory. The crosstalk between immune cells was explored using CellChat and the patterns of incoming and outgoing signals within the overall immune cell population were identified. Afterwards, the bulk RNA-sequencing data from The Cancer Genome Atlas (TCGA) were combined and the relative infiltration abundance of the identified subpopulations was analyzed using CIBERSORT. Moreover, cell composition patterns could be classified into five tumor microenvironment (TME) subtypes by employing a consistent non-negative matrix algorithm. Finally, the co-expression and interaction between SPP1+TAMs and Treg cells in the tumor microenvironment were analyzed by multiplex immunohistochemistry.

**Results:**

In the T cell lineage, we found that CXCL13+T cells were more widely distributed in colorectal cancer tissues, and the proportion of infiltration was increased. In addition, Th17 was found accounted for the highest proportion in CD39+CD101+PD1+T cells. Mover, Ma1-SPP1 showed the characteristics of M2 phenotypes and displayed an increased proportion in tumor tissues, which may promote angiogenesis. Plasma cells (PCs) displayed a significantly heterogeneous distribution in tumor as well as normal tissues. Specifically, the IgA+ PC population could be shown to be decreased in colorectal tumor tissues whereas the IgG+ PC one was enriched. In addition, information flow mediated by SPP1 and CD44, regulate signaling pathways of tumor progression. Among the five TME subtypes, the TME-1 subtype displayed a markedly reduced proportion of T-cell infiltration with the highest proportion of macrophages which was correlated to the worst prognosis. Finally, the co-expression and interaction between SPP1+TAMs and Treg cells were observed in the CD44 enriched region.

**Discussion:**

The heterogeneity distribution and phenotype of immune cells were analyzed in colon cancer and rectal cancer at the single-cell level. Further, the prognostic role of major tumor-infiltrating lymphocytes and TME subtypes in CRC was evaluated by integrating bulk RNA. These findings provide novel insight into the immunotherapy of CRC.

## Introduction

Colorectal cancer (CRC) is one of the most common malignant tumors in the digestive system and the third main cause of mortality related to malignant tumors worldwide (1, 2). CRC often generically refers to colon cancer and rectal cancer, but in fact, it is a tumor with high heterogeneity. Of note, the specificity of the proximal to distal intestinal development creates different microbial communities and gene and protein expression patterns among different regions in the intestine during development, resulting in various physiological functions (3). Consequently, colon and rectal cancers exhibit differences in pathological features, treatment regimens, and prognostic outcomes (4–6). Fortunately, the emergence of single-cell sequencing has enabled the analysis of inter- and intra-tumor heterogeneity (7, 8). Yet, no study has been conducted to elucidate the heterogeneity of immune cell subpopulations in the colon and rectal cancers with single-cell sequencing.

Breakthroughs in immunotherapy have been achieved in cancer treatments (9). Nevertheless, immunotherapy is not beneficial for all tumor patients, as the response to it largely depends on the characteristics of the tumor microenvironment (TME) (10). The efficacy of immunotherapy is remarkably affected by the intricate interaction between cytotoxic T cells and natural killer (NK) cells that play an important role in immune surveillance, as well as regulatory T cells (Tregs) and tumor-associated macrophages (TAMs) that dominate the immunosuppressive microenvironment (11, 12). In addition, the TME can be reprogrammed by the interconversion of T cells between the pre-exhausted and exhausted states and macrophages between the M1 and M2 phenotypes (13, 14).

Likewise, the prognosis of tumor patients and their responses to immunotherapy can be directly affected by the infiltration status and phenotypic heterogeneity of tumor-infiltrating immune cells, especially T cells, in solid tumor tissues (15). T cell dysregulation within tumors is progressive and dynamically process from pre-exhausted to terminal exhausted, which is considered an important factor affecting the efficacy of immunotherapy in CRC patients (16). Early dysfunctional T cells could regain effector function and reprogrammed into effector T cells through immunotherapy, which becomes a breakthrough to reverse the exhaustion of T cells, while late dysfunctional T cells cannot be saved due to their resistance to therapeutic reprogramming (17). PD1 expression increases with the progression of T cell exhaustion, in addition to the co-expression of CD38 and cd101 in late dysfunctional T cells may reflect fixed dysregulation of CD8^+^T cells, which indicates an adverse response to anti-PD1 immunotherapy (18). Whereas the co-expression of CD39 and CD103 has been suggested to be a marker of tumor antigen-specific TILs in solid tumors, in some researches including CRC, CD103^+^CD39^+^T cells have been suggested to be an immune marker to predict patient prognosis as well as response to ICB therapy (19–21). Therefore, identification of T cell heterogeneous phenotype could provide an aid for achieving effective patient stratification in immunotherapy.

Macrophages are highly heterogeneous cells, among which tumor-associated macrophages (TAMs) are important immune cells in TME (22). TAMs can promote tumorigenesis and metastasis and exert immunosuppressive effects through pro-angiogenesis, starvation of cytotoxic CD8+T cells, and recruitment of Tregs (23). Furthermore, TAM targeting has recently emerged as a hot spot in tumor immunotherapy. For instance, a prior study validated that CSF1R inhibitors could reduce TAMs in the TME and promote macrophage repolarization to M1, thus showing tremendous potential for clinical application (24). However, the specific heterogeneity of TAMs and the ambiguous time course of macrophage recruitment and polarization pose certain obstacles to TAM-targeted therapy (25). As key antigen-presenting cells (APCs), the two conventional subsets, cDC1 and cDC2, are responsible for the presentation of tumor-associated antigens to CD8+ T cells and CD4+ T cells, respectively (26, 27). Additionally, the two immunosuppressive APCs, plasmacytoid dendritic cells (pDCs) and novel mature LAMP3+ DCs, exist in the TME of various solid tumors and have attracted increasing attention (28–30). B cells, a major component of the TME, are also emerging as a key player in the anti-tumor immune response, whose function and distribution are highly dependent on tertiary lymphoid structures (TLSs) (31). Specifically, germinal center B cell clones in mature TLSs differentiate into plasma cells (PCs) that can produce IgG or IgA antibodies against tumor-related antigens (32). Importantly, B cells and TLSs have recently been demonstrated as the key to the clinical outcome of immunotherapy in tumor patients (33–36). Therefore, a deeper understanding of the immune cell landscape in the colon and rectal cancers and non-cancerous tissues can lay an essential theoretical foundation for achieving precision in immunotherapy for these diseases.

The large-scale single-cell CRC transcriptome database, created by integrating two published single-cell databases (GSE132465 and GSE146771) and describing an elaborate molecular signature of immune cells and the heterogeneity of TME in the colon and rectal cancers, was thoroughly investigated (37, 38). Importantly, our study also analyzed the crosstalk between immune cells with CellChat and identified patterns of incoming and outgoing signals of the overall immune cell population. Finally, we determined five TME immune cell infiltration patterns in CRC patients, and their relationships with CRC progression were investigated. Our research provides new insights into the immune microenvironment of CRC and provides new potential targets for CRC immunotherapy.

## Materials and methods

### Single-cell data processing and quality control

Firstly, 10x Genomics single-cell data were obtained from the SMC dataset of GSE132465 and the 10x Genomics dataset of GSE146771. Because CD45+ cells were isolated by fluorescence-activated cell sorting in advance, the 10x Genomics data of GSE146771 contained only the data of immune cells. Since only immune cells in tissues were analyzed in our study, blood cells in the GSE146771 dataset were removed and immune cells were extracted from the SMC dataset, followed by the merging of data from these two datasets using merge function. Then, 27 colon cancer tissues, 6 rectal cancer tissues, and 18 adjacent tissues were grouped according to the anatomical location provided in the clinical information table of the datasets for subsequent analyses. The single-cell RNA-sequencing data were created for Seurat objects with the Seurat package (4.1.0). Low-quality cells with unique feature counts > 6000 or < 300 or mitochondrial counts > 15%, as well as ribosomes < 3% and erythrocytes < 0.1%, were filtered, with 62,398cells remained after quality control.

## Unsupervised dimensionality reduction

A total of 62,398 immune cells were identified and classified into four major immune cell clusters (T cells, NK cells, B cells, and myeloid cells). These subpopulations were re-clustered into immune cell lineages. Specifically, the original metadata were normalized using the NormalizeData function, and the FindVariableFeatures function was used to select 2,000 hypervariable genes. Next, the data were scaled using the ScaleData function before the PCA dimension reduction. Next, the harmony function was used to remove the batch effect from the data. The FindClusters and cluster functions were used for cell reclustering. The resolution from 0.1 to 1 was used to obtain better sub-clusters. Potential marker genes were determined using the FindAllMarkers function and subjected to t-distributed Stochastic Neighbor Embedding (t-SNE) analyses. Typical marker genes were used to annotate cell clusters into known cell lineages.

### Pseudotime trajectory analysis

Monocle2 (version 2.20.0) was used for pseudotime analyses to determine the differentiation trajectory of cell development. After the UMI matrix was read from the Seurat object, the newCellDataSet function was utilized to create the object. Genes with mean expression > 0.1 were selected in the trajectory analysis, followed by dimension reduction with the DDRTree method and cell sorting with the orderCells function.

### SCENIC analysis

SCENIC (1.2.4) was used to analyze the enrichment of key transcriptomic factors in macrophage clusters. The motif Hg38 was selected as the SCENIC dataset, and 1500 cells were randomly selected to construct a co-expressed gene model. Next, the potential target genes of transcription factors were identified with GENIE3. DNA-motif enrichment analyses were performed with RcisTarget (1.14.0) to identify direct binding sites (regulons). The activity of each regulon in each cell was assessed with AUCell (1.16.0), followed by the calculation of the area under the receiver operating characteristic curve (AUC) and the integration of the expression rank of all genes in the regulon. The RegulonAUC matrix was imported into Seurat for the cluster analysis and visualization of single-cell data.

### Cell–cell communication analysis using CellChat

The intercellular communication between immune cell subsets in colon and rectal cancers was predicted with the Cellchat package (1.1.3) based on the analysis of ligand-receptor interactions. With the normalized Seurat data as the Cellchat object, CellChatDB.human was selected as the receptor-ligand interaction database. The communication probabilities were calculated with the computeCommunProb function to demonstrate cell interactions in terms of both the number and weight of interactions. The extractEnrichedLR function was utilized to extract all of the important interacting L-R pairs and related signaling genes of a given signaling pathway to present cell-cell communication mediated by a single L-R pair. In addition, global communication patterns and signal networks were analyzed with the CellChat adopted pattern recognition method based on non-negative matrix factorization (NMF).

### Gene set variation analysis pathway enrichment analysis

Hallmarks gene sets were downloaded from the Molecular Signatures Database (MSigdb). Afterwards, GSVA enrichment analyses were performed for cell subsets with the GSVA package (version 1.40.1). Additionally, the AverageExpression function was used to calculate the average gene expression in all cells of each subpopulation. The R package CluserProfiler (V4.0.5) was used for the pathway enrichment analysis of specific gene sets, with Adj.p.val < 0.05 considered significantly enriched pathways. Then, the key pathways were selected for visualization.

### Scoring of macrophage M1 and M2

The score of the macrophage subgroups M1 and M2 phenotypes referred to the average normalized expression of the characteristic genes related to classically activated M1 macrophages (SOCS1, NOS2, TNF, CXCL9, CXCL10, CXCL11, CD86, IL1A, IL1B, IL6, CCL5, IRF5, IRF1, and CCR7) and alternatively activated M2 macrophages (IL4R, CCL4, CCL18, CCL22, MARCO, VEGFA, CTSA, CTSB, TGFB1, MMP9, CLEC7A, MSR1, IRF4, CD163, TGM2, and MRC1).

### Distribution and proportion of CD39+CD101+PD1+T cells in CRC

If the cells conformed to the condition that the gene expression of CD39, CD101, or PD1 was > 0, they were defined as positive for the target gene. If the simultaneous expression of two or three target genes was > 0 at the same time, the cells were defined as double- or triple-positive. Only cells that simultaneously met the requirement of the expression of CD39, CD101 or PD1 being 0 were counted as triple-negative cells. The proportion of cells meeting these conditions was counted, and t-SNE was used to visualize the distribution of cells with different phenotypes.

### Cell subtype deconvolution based on bulk RNA-sequencing data and tumor microenvironment classification

The gene expression matrix was generated based on single-cell RNA (scRNA) sequencing (seq) data to characterize cell clusters. The CIBERSORT deconvolution algorithm was used to assess the relative infiltration abundance of each cell cluster in the colon adenocarcinoma (COAD; 480 tumor samples and 41 normal samples) and rectum adenocarcinoma (READ; 167 tumor samples and 10 normal samples) cohort from The Cancer Genome Atlas (TCGA). Then, the difference in the obtained relative infiltration abundance between tumor and normal tissues was calculated with the Wilcoxon test. The ConsensusClusterPlus package (1.58.0) (39) was utilized to determine the optimal K value and identify the cellular subtypes.

### Clinical sample collection

Approved by the Ethics Committee of the General Hospital of the Northern Theatre Command, PLA, China, we collected 9 tumor cases and adjacent normal tissues from 9 patients with the pathological diagnosis of CRC during surgical resection. All patients were diagnosed with primary colorectal tumors and were treatment Naïve. They ranged in age from 31 to 67, with a median age of 58. The clinical characteristics of these patients, including age, gender, pathological classification and stage, are shown in **Table S1**. The tissues were embedded in paraffin and sectioned at 4 μ M for subsequent immunofluorescence assay.

### The multiplex immunohistochemistry

We performed multiple immunohistochemical staining according to the kit manufacturer’ s instruction (Wuhan Powerful Biotechnology Co., LTD). In brief, after multiple rounds of repeated antigen repair, incubation of primary antibody, HRP labeling of secondary antibody and amplification of TSA fluorescence signal, a paraffin section was marked with multiple target fluorescence labeling, and finally DAPI was used to re-stain the nucleus. Spectral imaging was performed with a multi-spectral tissue imaging system (FI3, Nikon, Japan), followed by Image scanning and analysis using caseviewer and Image J. The antibodies used in the experiment were as follows IgA (Abcam, ab124716), IgG (Abcam, ab109489), CD163 (Abcam, ab182422), CD44 (Abcam, ab6124), SPP1 (Osteopontin) (Abcam, ab214050), pan Cytokeratin (Abcam, ab7753), Foxp3 (Abcam, ab215206).

## Statistical analysis

Statistical analysis was performed with R 4.2.3, SPSS v26, and Prism 8.0. Data with normal distribution were compared by the 2-tailed Student’s t-test, and data with abnormal distribution were compared by Wilcoxon’s rank-sum test. The Kruskal-Wallis test, a nonparametric test, was utilized for comparisons among three or more independent groups. Survival was analyzed with the Kaplan-Meier method and log-rank test.

## Results

### Single-cell profiling of immunogenomic landscape in the microenvironment of CRC

**Figure 1:**
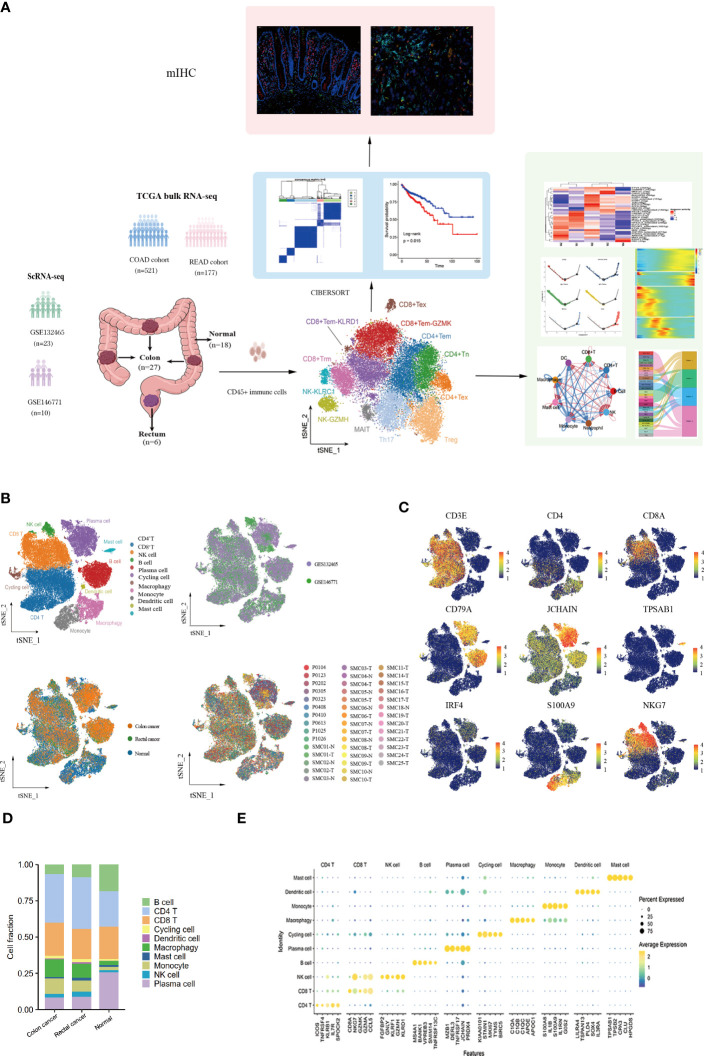
Landscape of immune cells in the microenvironment of colon cancer, rectal cancer, and adjacent normal tissues at the single-cell transcription level. **(A)**Schematic diagram explaining the workflow of the experimental design. **(B)** t-SNE (t-distributed Stochastic Neighbor Embedding) plot of 62,398 high-quality immune cells showing the major immune cell type clusters in the tumor microenvironment (TME) of colorectal cancer (CRC) and adjacent normal tissues, color-coded by dataset, tissue types, and patients. **(C)** t-SNE plots showing the expression levels of representative marker genes for major immune cell clusters, color-coded by gene expression levels. **(D)** Stacked bar plots showing the cell fractions of major immune cell types in colon cancer, rectal cancer, and adjacent normal samples. **(E)** Bubble plot showing the average expression level of the top 5 marker genes for the 10 major immune cell clusters.

10x scRNA-seq data were obtained from GSE132465 and GSE146771 datasets, including 18 adjacent normal samples (10 from GSE132465 and 8 from GSE146771), 27 colon tumor samples (20 from GSE132465 and 7 from GSE146771), and 6 rectal tumor samples (3 from GSE132465 and 3 from GSE146771). The clinical information of all patients is detailed in **Table S2**. A schematic chat of the experimental design was showed in **Figure 1A**. These two datasets were integrated by removing the batch effect of samples, and the obtained major cell types were derived from different patients with low patient specificity. After quality control and filtration, 62,398 immune cells were retained for unsupervised clustering, including 24,698 cells from adjacent normal tissues, 30,579 cells from colon cancer tissues, and 7,121 cells from rectal cancer tissues. 10 major immune cell subpopulations, including CD8^+^ T cells, CD4^+^ T cells, NK cells, B cells, plasma cells, cycling cells, macrophages, monocytes, dendritic cells (DCs), and mast cells, were successfully identified according to their typical marker genes using the T-distributed randomly adjacent embedding (t-SNE) dimension reduction method. Cells stemming from different datasets and tissues were classified and color-coded. All cellular subgroups were evenly distributed resulting in no obvious patient- or disease-specific pattern (**Figure 1B**). The typical marker genes for each cluster were visualized using t-SNE plots and the top5 genes were displayed using the bubble plot (**Figures 1C, E**). T lymphocytes are the main tumor-infiltrating immune cells in the TME of CRC however, the proportion of total T lymphoid lineage cells did not display differences between tissues. We found that the B lineage had a decreased proportion in CRC samples compared to normal tissues, conversely, myeloid cells had a higher proportion in tumor tissues (**Figure 1D**). Taken together, we performed an unsupervised re-clustering of major immune cell subpopulations to comprehensively explore the heterogeneity of the colorectal cancer microenvironment.

### Characterization of the heterogeneity of T and NK cell subtypes in CRC

**Figure 2:**
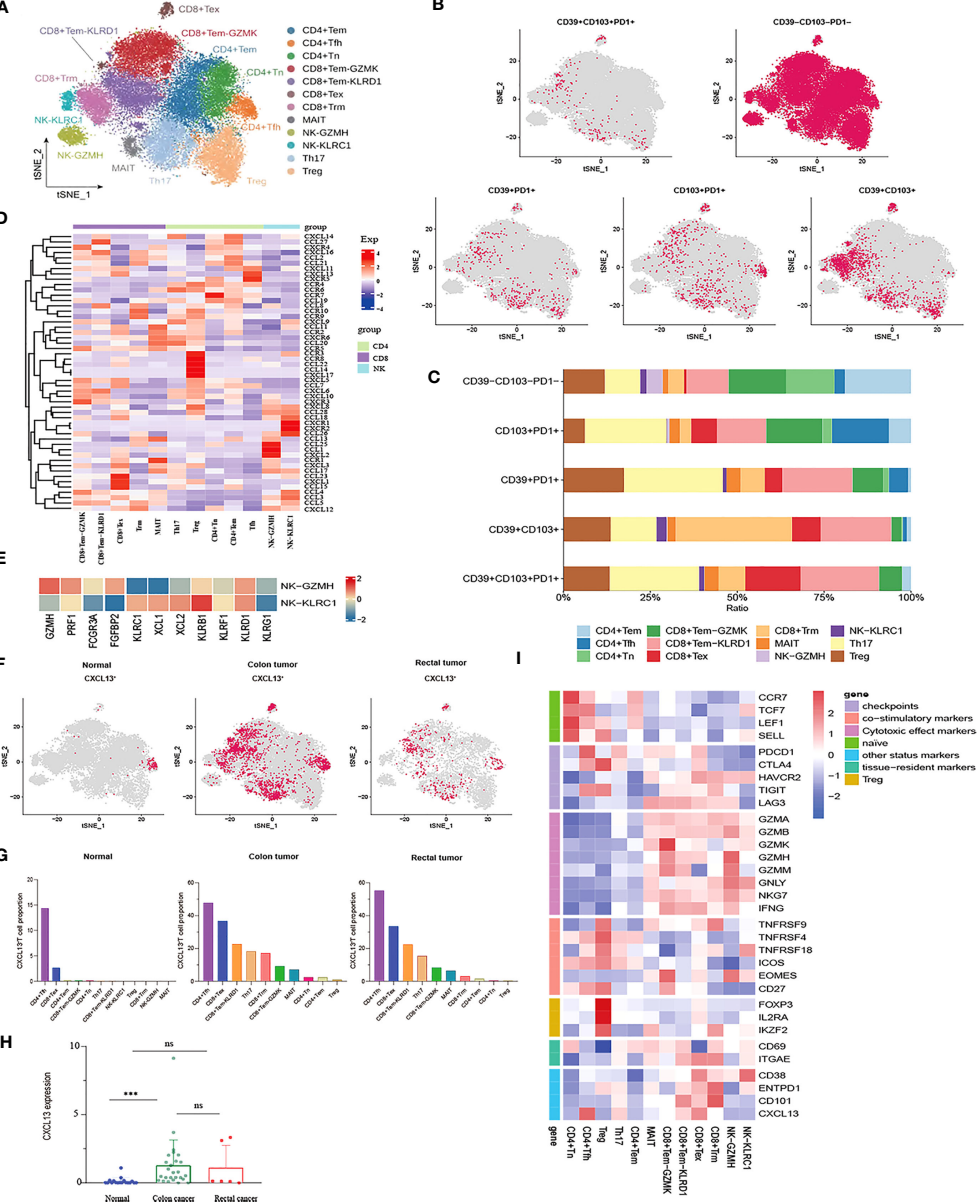
Characterization of phenotypes of T cells and NK cells in colon cancer, rectal cancer and adjacent normal tissues. **(A)** t-SNE plot of a total of 32,879 T cells re-clustered into 10 clusters and 1,898 NK cells re-clustered into 2 clusters using color-coded by cell type. **(B)** t-SNE projections showing the expression and distribution of phenotypic marker genres CD39, CD103, and PD1 in T andNK clusters. Each dot represents a cell defined as positive expression of marker genes. **(C)** Stacked bar plots displaying the percentages of each cluster from T and NK subpopulations with different CD39, CD103, and PD1phenotypes. **(D)** Heatmap showing the expression profile of canonical chemokines and chemokine receptors of T and NK clusters. **(E)** Heatmap demonstrating the expression characteristics of special functional genes in two NK subgroups with different phenotypes **(F)** t-SNE projections of CXCL13 expression distribution in normal, colon and rectal tissues. **(G)** Box plot comparing the expression levels of CXCL13 in T andNK lineage among calculated using Kruskal-Wallis test, ***p< 0.001, ns p > 0.05. **(H)** Fractions of CXCL13^+^ cells among T andNK subpopulations in different tissues from scRNA-seq datasets. **(I)** Heatmap of the relative expression of function related genes, including naïve, cytotoxic, exhaustion, co-stimulatory and resident in T andNK cell subsets.

After the unsupervised clustering, the obtained 32,879 T cells were classified into five CD4^+^ clusters (CD4^+^ Naïve, CD4^+^ Tem, Tfh, Th17, and Treg) and five CD8^+^ clusters (CD8^+^ Tem-KLRD1, CD8^+^ Tem-GZMK, Trm, MAIT and CD8^+^ Tex) (**Figure 2A**). Naïve CD4^+^ T cell cluster exhibited the high expression of Naïve T cell marker genes CCR7, TCF7, and SELL. However, no Naïve CD8^+^ T cells were identified. Additionally, a subpopulation of Memory CD4^+^ T cells was identified, which was characterized by the expression of ANXA1, GPR183, and IL7R (**Supplementary Figure 1A**). No cytotoxic genes and exhaustion marker genes were found to be highly expressed in the CD4^+^ Tem cluster (**Figure 2I**). According to the phenotypes of effector molecules, two effector memory CD8^+^T cells were identified: CD8^+^Tem-GZMK with high expression effector molecule GZMK, and the CD8^+^ Tem-KLRD1 cluster was characterized by the high expression of the NK cell inhibitory receptor KLRD1 Meanwhile, GZMK was almost not expressed in the CD8^+^ Tem-KLRD1 cluster, and the effector molecules NKG7, GZMA and IFNG were highly expressed in both subclusters with higher expression in the CD8^+^Tem-GZMK cluster, suggesting a predominance of cytotoxicity (**Supplementary Figure 1C**, **Figure 2I**).

The CD8^+^ Tex cluster exhibited the high expression of exhaustion markers including HAVCR2, PDCD1, CTLA4, and LAG3. The majority of effector molecules such as GNLY, GZMK, GZMA and NKG7 were also expressed in the CD8^+^ Tex cluster. Furthermore, co-expression of CD38/CD101 is a marker of terminal exhaustion T cells. In this study, CD38 and CD101 were expressed at higher levels in the CD8^+^ Tex cluster, suggesting that the CD8^+^ Tex cluster was in a state of terminal exhaustion (**Figure 2I**). Likewise, the proliferation- and cell cycle-related genes MKI67, STMN1, and TOP2A were also upregulated in the CD8^+^ Tex cluster, indicating that certain CD8^+^ Tex cells were in a proliferative state (**Figure 2B**). In fact, prior studies have confirmed exhausted CD8^+^ T cells as the highly proliferating cell population in the TME at a specific stage (40, 41). In addition, the Treg cluster characteristically expressed the marker genes FOXP3 and IL2RA while highly expressed the T cell co-stimulatory factors TNFRSF4, TNFRSF18, TNFRSF9, CD27, and ICOS, especially TNFRSF9, which is a known activation marker for antigen-specific Tregs. Of note, the Treg cluster had higher expression of CTLA4 and TIGIT than any other exhausted T cell clusters (**Figure 2I**). Therefore, it could be concluded that the Treg cluster might play a pivotal role in the immunosuppression of CRC due to its higher infiltration level in tumor tissues and lower infiltration level in normal tissues.

A unique class of unconventional mucosal associated invariant (MAIT) cells with a profile of the marker genes KLRB1 (CD161), NCR3, RORA, and SLC4A10 and the inhibitory NK cell receptors KLRG1, KLRB1, and IL4I1 (a tumor-derived tryptophan catabolic enzyme that promotes tumor invasion) exists in the intestinal mucosal tissues. Likewise, the MAIT cluster was also noted to characteristically overexpress CEBPD, a transcription factor associated with a variety of malignancies (**Supplementary Figure 2C**). Intriguingly, the MAIT cluster maintained the activity of cytotoxic effectors. Meanwhile, PDCD1, CTLA4, HAVCR2, and LAG3 were differentially expressed, among which LAG3 was the most significantly expressed, but CD38 and CD101 were not expressed in this cluster (**Figure 2I**). Therefore, we speculated that the MAIT cluster might be a kind of T cell in an exhausted state but might be in a pre-exhausted state compared with the CD8^+^ Tex cluster.

A CD8^+^ T cell cluster characterized by high expression of ITGAE (CD103) and CD69 represented tissue-resident memory T (Trm) cells, which permanently reside in tissues instead of returning to the blood circulation and can mediate rapid immune responses. Trm cells exert an immunosurveillance effect, which has been implicated in preventing the development of solid tumors. In the Trm cluster, the cytotoxic factors GZMA, GZMB, NKG7, and GNLY were upregulated. Trm cells have been verified to still maintain the ability to produce cytotoxic molecules and effector cytokines despite the high expression of the immune checkpoints HAVCR2, LAG3, and TIGIT in these cells, suggesting that CD103^+^ Trm is more resistant to exhaustion than circulating T cells. Unlike CD8^+^ Tex, Trm cells can restore immune function when re-exposed to appropriate antigens. Moreover, ENTPD1 (CD39) was higher expression in the Trm cluster, demonstrating that Trm cells were reactive tumor-infiltrating lymphocytes (TILs) distinct from bystander T cells (**Figure 2I**).

CD39, CD103, and PD-1 have been independently considered markers of tumor-reactive CD8^+^ TILs (20). Co-expression of PD-1, CD103, and CD39 is crucial for stratifying patients receiving immunotherapy. It is generally believed that CD39, CD103, and PD-1 are co-expressed, and triple-positive TILs may be related to improved response rates and prognostic outcomes (42, 43). Nevertheless, our study found that PD1^+^CD39^+^CD103^+^ T cells were rarely observed in CRC and that these small numbers of triple-positive cells were mainly distributed in the Th17 and CD8^+^Tem-KLRD1 subsets. Similarly, compared with other T cell subpopulations, Th17 and CD8+ Tem-KLRD1 cells accounted for a higher proportion of PD1^+^CD103^+^ T and CD39^+^PD1^+^ T cells, and CD8^+^Trm accounted for the highest proportion of CD39^+^CD103^+^ T cells (**Figures 2B, C**). Different phenotypes of Th17 cells, especially CD39^+^CD101^+^PD1^+^ Th17 cells, may be critical for the prediction of tumor-reactive TILs; however, such studies are currently lacking. We also found that PD1 was poorly expressed in tissue-resident T cells, whereas HAVCR2 was highly expressed (**Figure 2I**). Therefore, we speculate that this type of TRM might respond better to anti-TIM3 treatment.

The 1,898 NK cells were mainly divided into two clusters: the NK-KLRC1 cluster expressing the inhibitory receptors KLRC (NKG2A) and KLBR1; the NK-GZMH cluster expressing the inhibitory receptors KLRD1, as well as cytotoxic molecules GZMH and PRF1. Meanwhile, the NK-GZMH cluster had a high level of the exhaustion markers HAVCR2 and LAG3, while the NK-KLRC1 cluster expressed HAVCR2 and CD38, indicating that both NK cell clusters were representative of a certain degree of exhaustion (**Figure 2I**). Furthermore, CD16 and FGFBP2 (CD14) were highly expressed in the NK-KLRC1 cluster and, conversely, poorly expressed in the NK-GZMH cluster (**Figure 2E**). It is widely recognized that most NK cells in the blood have the characteristics of CD16^+^ FGFBP2^+^ (CD14^+^), so it is hypothesized that the NK-KLRC1 cluster infiltrated in tissues is derived from NK cells in the blood. Meanwhile, chemokines were identified to show different expression levels between the two NK clusters, among which XCL1 and XCL2 were the main chemokines expressed in the less cytotoxic NK-KLRC1 cluster (**Figure 2E**). Importantly, these two chemokines are extensively accepted to recruit XCR1^+^ cross-presenting DCs into the tumor to cause tumor-driving inflammation, thus changing a “cold tumor” into a “hot tumor”.

Cluster analysis was performed on the expression patterns of major chemokines and receptors of T and natural killer (NK) cell subgroups in the tumor microenvironment of colorectal cancer. The results indicated that the CD8^+^Tex subgroup highly expressed chemokines that promoted angiogenesis and participated in the migration of inhibitory immune cells, such as CCL15, CXCL1, and CXCL12. In addition to the well-known chemokine receptor CCR4, the Treg cell subset also higher expression CCL22, CCL14, CXCL17, CCR3, and CCR8. Chemokines CCL24, CCL1, and CXC12 were highly expressed in the NK-GZMH subgroup, while chemokine CCL26 and chemokine receptors CXCR1 and CXCR2 were highly expressed in the NK-KLRC1 subgroup. The CD8^+^Tem GZMK subgroup highly expressed CXCR3, and the chemokines CXCL19, CXCL10, and CXCL11 interacted with the immune cells of CXCR3^+^ to recruit cells with anti-angiogenesis function. The follicular helper T (Tfh) cell subgroup higher expression chemokines CXCL11, CXCL13, and CXCR5 (**Figure 2D**). The CXCL13-CXCR5 axis can induce tumorigenicity or anti-tumor immune response in the tumor microenvironment by recruiting multiple lymphocyte populations. On the one hand, the CXCL13 signal plays a leading role in the recruitment of B cells and the formation of tertiary lymphoid structures, activating the immune response of some tumors (44); on the other hand, CXCL13 is critical for driving the occurrence, development, and metastasis of malignant tumor (45). We analyzed the expression and distribution of CXCL13 in the tumor microenvironment of CRC (**Figure 2F**). Compared with that in normal tissues, the expression of CXCL13 in colon cancer tissues was significantly increased, but there was no significant difference in rectal cancer tissues (**Figure 2H**). The distribution of CXCL13^+^ T cells in normal, colon, and rectal cancer tissues had significant heterogeneity. In normal tissues, CXCL13^+^ T cells were mainly distributed in CD4^+^ Tfh and CD8^+^ Tex subgroups. However, CXCL13^+^ T cells showed a wider distribution in cancer tissues. In addition to CD4^+^ Tfh and CD8^+^ Tex subsets, CXCL13^+^ T cells also had a high proportion in Th17 and CD8^+^ Tem subsets in cancer tissues (**Figure 2G**). We speculated that the differential expression of CXCL13 in different cell subsets may play distinct roles in tumor progression and immune promotion.

### Landscape of the heterogeneity and diversity of myeloid cell in the TME of CRC

**Figure 3:**
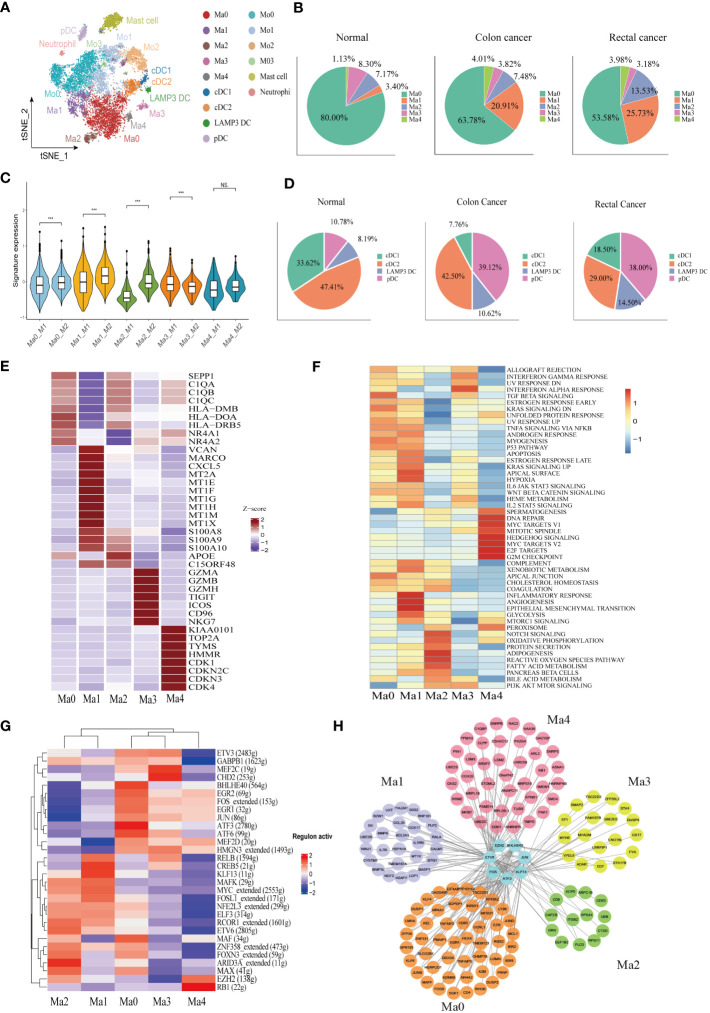
Identification of the heterogeneity of myeloid cells in colon cancer, rectal cancer, and adjacent normal tissues. **(A)** t-SNE plot of 10,514 myeloid cells re-clustered into 15 clusters. **(B)** Pie chart presenting the proportion of five different phenotypes macrophage in the whole macrophage lineage. **(C)** Violin plot comparing the scores of M1 and M2 macrophage clusters by wilcox. test, ***p < 0.001, ns p > 0.05. **(D)** Pie chart presenting the proportion of four different dendritic cell (DC) subtypes in the whole DC lineage. **(E)** Heatmap showing the key differentially expressed genes (DEGs) of each macrophage cluster. **(F)** Heatmap showing the enriched pathways from hallmark gene sets in macrophage clusters using gene set variation analysis (GSVA). **(G)** Heatmap of the top 30 regulators with the highest area under curve (AUC) scores showing the activity of transcription factors (TFs) in macrophage clusters using single-cell regulatory network inference and clustering (SCENIC). **(H)** Protein-protein interaction (PPI) networks of prominent TF-target genes in 5 macrophage clusters.

A total of 10,514 macrophages underwent unsupervised re-clustering into five macrophage clusters (3,748), four monocyte clusters (4,925), three DC clusters (1,025), mast cell cluster (1,025) and neutrophils (95) (**Figure 3A**). The Ma0 subgroup was characterized by SEPP1 expression, in which the complement pathway-related genes such as C1QA, C1QB, and C1QC were prominently expressed and the orphan nuclear receptors (NR4A1, NR4A2, and NR3A3) that mediate macrophage-induced inflammation were up-regulated. Furthermore, the abundant expression of MHC II molecules in this cluster indicated that SEPP1^+^ TAM possessed a strong ability of antigen presentation (**Figure 3E**). Among the hallmark gene sets for Gene Set Variation Analysis (GSVA), TGFβ and KRAS signaling pathways were found to be enriched mainly in the Ma0 subgroup (**Figure 3F**).

The Ma1 cluster characteristically expressed SPP1, as well as high expression of marco which can promote M2 macrophage polarization, meanwhile CXCL5, which promotes tumor metastasis, was also highly expressed in this subpopulation. Likewise, metallothionein including MT2A, MT1E, and MT1F was abundantly expressed in this cluster, therefore assuming a crucial role in the formation, progression, and drug resistance of tumors. In addition, S100A proteins were also upregulated in the Ma1 subgroup (**Figure 3E**, **Supplementary Figure 2A**). Ma1 cells were involved in angiogenesis, epithelial mesenchymal transformation and inflammation-related signaling pathways (**Figure 3F**) Ma2 had higher APOE expression than other macrophage subtypes (**Figure 3E**, **Supplementary Figure 2A**), which was mainly related to lipid metabolism and reactive oxygen species (**Figure 3F**). Ma3 exhibited T cell gene profile with highly expressive of T cell signature genes, which were associated with interferon-α and interferon-γ response pathways (**Supplementary Figure 2A**, **Figure 3F**). Ma4 was with the characteristic high expression of the proliferation-related genes KIAA0101, TOP2A and MKI67 and the abundant expression of the cell cycle-related genes (CDK1, CDKN2C, CDKN3, and CDK4) (**Figure 3E**. The results of GSVA demonstrated that the cluster was located mainly in DNA repair, MYC and cell cycle related signaling pathways (**Figure 3F**). Macrophage clusters were scored based on the average expression of M1 and M2-like signature genes. The results showed that Ma0, Ma1, and Ma2 tended to be M2-like phenotypes and Ma3 cluster was inclined to the M1-like phenotypes, while Ma4 had no significant difference in the two phenotypes (**Figure 3C**).

Analysis of the proportion of each macrophage subgroup showed that Ma0 had the largest proportion, and Ma0 was the dominant macrophage subgroup in both normal and CRC tissues. In normal paracancerous tissues, Ma3 was the main macrophage subgroup, except for Ma0; however, in CRC tissues, Ma1 was the dominant subgroup, except for Ma0, and Ma3 accounted for the lowest proportion (**Figure 3B**). The proportions of macrophage subgroups in myeloid cells from various tissues were further analyzed. Compared to normal tissues, colon cancer tissues had a significantly increased proportion of Ma1 and Ma4 but only a slightly increased proportion of Ma2. There were no significant differences in the proportions of Ma0 and Ma3 in the various tissues. However, there was no significant difference in the proportion of macrophages in various rectal cancer subtypes compared to that in colon cancer or normal tissues. (**Supplementary Figure 2I**). Therefore, we speculate that Ma1 is a major tumor-associated macrophage with immunosuppressive effects in colon cancer.

SCENIC was used to analyze the key transcription factors (TFs) that may regulate macrophages, followed by identification of the top 30 TFs ranked by the relative activity scores of regulons. The results showed that two important TAM-related transcription factors, BHLHE40 and ATF3, were highly expressed at Ma0. BHLHE40 promotes the expression of proinflammatory genes in macrophages (46), while ATF3 negatively regulates macrophages (47). FOS and JUN proto-oncogenes were also upregulated in the Ma0 cell subsets. CREB5 and KLF13, which influence macrophage polarization, were highly expressed in the Ma1 cluster. The Kruppel-like Factor (KLF) family is vital for regulating macrophage-mediated inflammation (48). For example, KLF13 knockdown downregulates the expression of M1 macrophage-related factors induced by lipopolysaccharides (49). In addition, ARID3A, a gene involved in the maturation of macrophages and the promotion of M2 macrophage polarization (50), was highly expressed in the Ma2 cluster (**Figure 3G**). A protein–protein interaction (PPI) network was constructed for key regulatory transcription factors and core target genes of the macrophage subgroups. The results showed that the number of interactions between transcription factors and target genes in Ma0 was the largest and that the target genes were jointly regulated by multiple TFs (**Figure 3H**).

Monocytes were further re-clustered into four clusters as per CD14 and CD16 expression: Mo0 (CD14^++^CD16-), Mo1 (CD14^++^CD16^+^), Mo2 (CD14^+^CD16^-^), and Mo3 (CD14^+^CD16^+^) (**Supplementary Figure 2C**). Mo0 stimulated the release of cytokines and chemokines such as IL6, IL1A, CXCL1, CXCL5, and CCL20, which can induce monocyte recruitment to tumor. In addition, high expression of INHBA is thought to promote the proliferation of colon cancer cells (51). Mo1 had different gene expression patterns from Mo0, and some chemokines, CXCL9, CXCL10 and CXCL11, were significantly increased in this subgroup. In addition, IFN induction genes IFIT2 and IFIT3 are highly expressed. We also found significant upregulation of IDO1 expression in this subpopulation. Mo2 subgroup expressed macrophage characteristic genes, such as complement pathway related genes C1QA, C1QB, C1QC, and lipid metabolism related genes APOE and APOC1(**Supplementary Figure 2D**).

Dendritic cells (DCs) were re-clustered into four clusters according to the characteristic marker genes, including cDC1 (CLEC9A and BATF3), cDC2 (CLEC10A, CD1C, and FCER1A), pDC (CLECA4, IL3RA, and LILRA4), and LAMP3^+^ DC (CCR7 and LAMP3) (**Supplementary Figure 2B**). We identified a mature DC cell, LAMP3 ^+^DC, in CRC, which is believed to play a role in tumor cell migration in a variety of cancers. LAMP3^+^DC highly expresses chemokine CCL19 and its receptor CCR7, which may recruit other immune cells to migrate to tumor tissues through the CCL19-CCR7 axis. In addition, IDO1 was found to be significantly higher expression in LAMP3^+^DC (**Supplementary Figure 2G**). GSVA results indicated that the LAMP3^+^ DC cluster was related to IL2, IL6, and interferon signaling pathways (**Supplementary Figure 2F**). Plasmacytoid dendritic cells (pDCs) comprise a subset of dendritic cells characterized by the ability to participate in inflammatory responses and exert immunosuppressive actions. However, we found that GZMB was abundantly expressed in the pDC subgroup (**Supplementary Figure 2G**). Consistently, previous studiesalso confirmed that pDCs were the main source of GZMB. The high expression of GZMB in pDCs may be induced by interleukin. The cytotoxicity function of this derived GZMB is inferior to its immune regulation. Hence, it does not directly induce apoptosis of target cells, but the proliferation of T cells can be inhibited in a GZMB-dependent manner (52, 53). The pDC cluster correlated to DNA repair, KRAS, mTOR, and CRC signaling pathways (**Supplementary Figure 2F**). The proportion of the pDC subgroup was increased significantly in cancer tissues, while the proportion of cDC1 in colon cancer DC cells was significantly reduced (**Figure 3D**). The analysis of DC proportion in various tissues showed that the proportion of pDCs in colon cancer and rectal cancer tissues was increased to a certain extent compared with that in normal tissues (p < 0.05), while the proportion of cDC1 in colon cancer tissues was decreased significantly (p < 0.001). However, there was no significant difference in LAMP3^+^ DC proportion among groups (p > 0.05) (**Supplementary Figure 2I**).

### Identification of landscape of B lymphocytes and developmental trajectory states of B lineage of CRC

**Figure 4:**
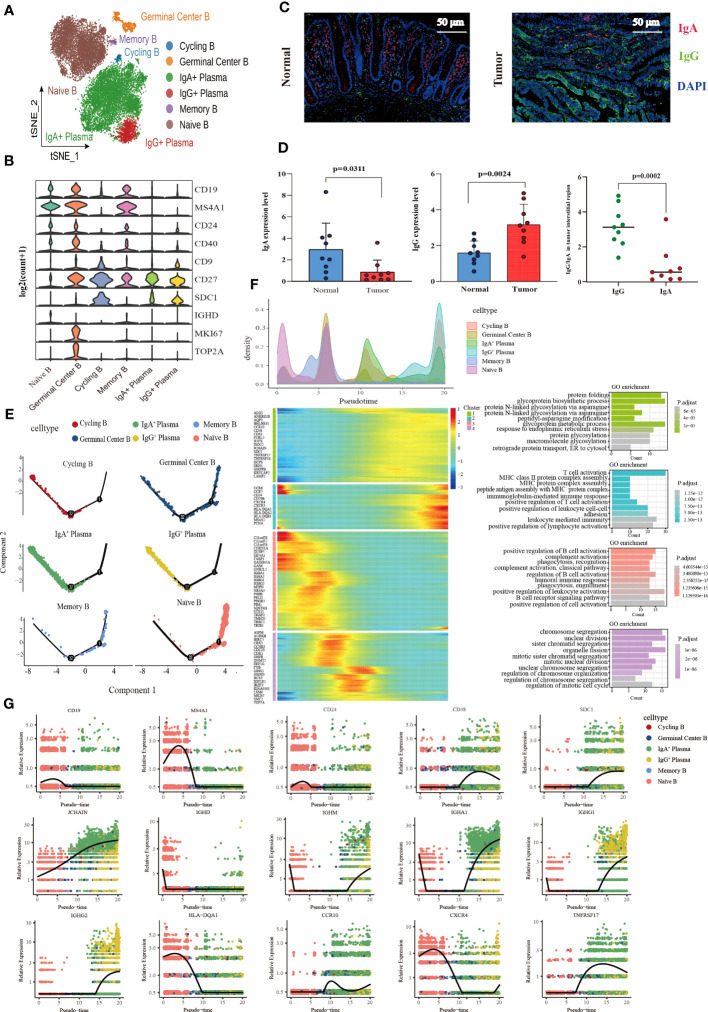
Characterization of the landscape of B lymphocytes and developmental trajectories of B lineage in CRC. **(A)** t-SNE projections of 17,107 B cells re-clustered into 6 major clusters. **(B)** Violin plot of relative expression of key characteristic genes in B lineage clusters. **(C)** Representative images of fluorescence staining showing the expression and distribution of IgA and IgG in normal intestinal tissue (left) and CRC tissue (right), respectively. Red representing IgA, green representing IgG, and blue representing DAPI, scale bar=50μm. **(D)** Statistical analysis results of immunofluorescence staining indicating the average expression of IgA decreased in CRC tissues compared with normal intestinal epithelium (left), whereas the average expression of IgG increased (middle), and the expression of IgG higher than that of IgA in tumor stroma (left). **(E)** Developmental trajectories of B lineage inferred using monocle2, each cell subtype marked with a different color. **(F)** Cell density variation of B cell subtypes during the pseudotime (top), pseudo-heatmap of the representative DEGs in differentiation branches (left bottom), Gene Ontology (GO) functional enrichment analysis of DEGs re-clustered into 4 clusters (right bottom). **(G)** Pseudo-scatter plots showing the expression variation and distribution of some specific genes during the pseudotime, color-coded by cell types.

A total of 17,107 B cells were determined and then clustered with unsupervised clustering into six clusters, Naïve B cell cluster (6,849), germinal center B cell cluster (671), two plasma cell clusters IgA^+^ plasma cell cluster (8,190) and IgG^+^ plasma cell cluster (1,118), one memory B cell cluster (131), and one cycling B cell cluster (148) (**Figure 4A**). We found that the infiltration of plasma cells (PCs) was significantly heterogeneous among different tissues. Compared with normal tissues, the infiltration abundance of IgA^+^ PCs in CRC decreased. In contrast, the proportion of IgG^+^ PC was elevated significantly in colon cancer tissues compared to normal tissues but not statistically significant increase in the rectal cancer group (**Supplementary Figure 3A**). Meanwhile, analysis of B cell DEGs among different tissues revealed that IgG-related gene (IGHG1-4) was highly expressed in colon cancer (**Supplementary Figure 3B**). The results of immunofluorescence also verified that IgA was mainly enriched in the mucosal layer of normal tissue, while IgG was more abundant in the intermuscular stroma of normal tissue. The average expression level of IgA in normal tissue was higher than that of IgG. On the contrary, in cancer tissues, IgG enrichment was observed, but not IgA (**Figure 4C, D**).

In terms of molecular phenotype, PCs and Cycling B were featured by low expression of CD19 and MS4A1 (CD20). In additional, PCs showed CD138^+^CD27^+^CD38^+^ phenotype. CD24 was poorly expressed in the IgA^+^ PCs, while it expressed in the IgG^+^ plasma cluster (**Supplementary Figure 3C**). Cycling B cell cluster was named according to previous studies (54), which exhibited the characteristics of both B and T cells and the upregulation of the effector molecules of T cells including KLRB1, ANXA1, NKG7, GZMA and IL7R. Furthermore, the memory B cell cluster also expressed T cell signature genes and effector molecules, with a similar high expression gene pattern to the cycling B cell cluster. Interestingly, these two clusters were noticeably different regarding B cell signature genes. The memory B cell cluster had the high expression of CD19 and MS4A1 (CD20) and FCER2 (CD23) and the low expression of CD38, SDC1 (CD138), and IgA (IGHA1 and IGHA2) and IgM (IGHM) related genes, which was contrary to the cycling B cell cluster. More importantly, IGHM expression was significantly higher in the cycling B cell cluster than in other clusters, indicating that cycling B cells were immature B cell (**Supplementary Figure 3D**). Additionally, both the Naïve B and Germinal Center B cell clusters were characterized by the high expression of CD19, MS4A1 (CD20), CD24, and CD40 and the low expression of CD27, and SDC1 (CD138). The germinal center B cell cluster characteristically expressed the high level of the proliferation-related genes MKI67 and TOP2A (**Figure 4B**).

The pseudotime trajectory analysis of B cell clusters was performed with Monocle2 to elucidate the B cell developmental trajectory, as well as the distribution of branches and the cell density of each cluster. Among these clusters, naïve B cells were located at the beginning of the branch and subsequently differentiated to memory B cells. Germinal center B cells were distributed throughout the trajectory and eventually differentiated to IgA^+^ PCs, IgG^+^ PCs and Cycling B cells at the end of the trajectory (**Figure 4E**). A total of 459 differential genes were yielded through the pseudotime trajectory analysis and allocated to 4 clusters based on gene classification with similar patterns. As reflected by the results of GO enrichment analyses, genes in Cluster 1 were mainly enriched in the signaling pathways that modulate protein biosynthesis, genes in Cluster 2 and 3 majorly in the signaling pathways involved in the immune response process, and genes in Cluster 4 primarily related to the signaling pathways implicated in cell cycle processes (**Figure 4F**). The pseudotime dynamic changes of key genes during the development of B cells were analyzed. The results revealed that CD19, MS4A1 and CD24 were mainly expressed in Naïve B cells at the early stage of development and gradually decreased with the development of B cells, while CD38 was mainly expressed in PCs at the late stage of development and increased first and then decreased with time. In addition, CD138 was also expressed in PCs, and its expression gradually increased with time. Immunoglobulin-related genes were rearranged during B cell development. IGHD was mainly expressed in the early development of B cells and gradually disappeared with the activation of B cells. JCHAIN was distributed throughout the development of B cells, and its expression gradually increased with time. It is generally believed that the differentiation from B cells to PCs undergoes a process of switching from IgM to IgG. However, we found that IGHM expression rapidly decreased and disappeared at the early stage of B cell development, and then gradually increased in the late development of B cells. MHC II molecules were highly expressed at the early stage of B cell development and eventually disappeared during the development of PCs. During the development of B cells, the expression of CCR10 changed from low to high, and the expression of CXCR4 decreased gradually. TNFRSF17, a marker of B lymphocyte maturation, was mainly expressed in mature B cells and PCs, which played a vital role in B cell maturation and autoimmune response (**Figure 4G**).

### Evaluation of the infiltration abundance and prognostic value of the major cell subpopulations

**Figure 5:**
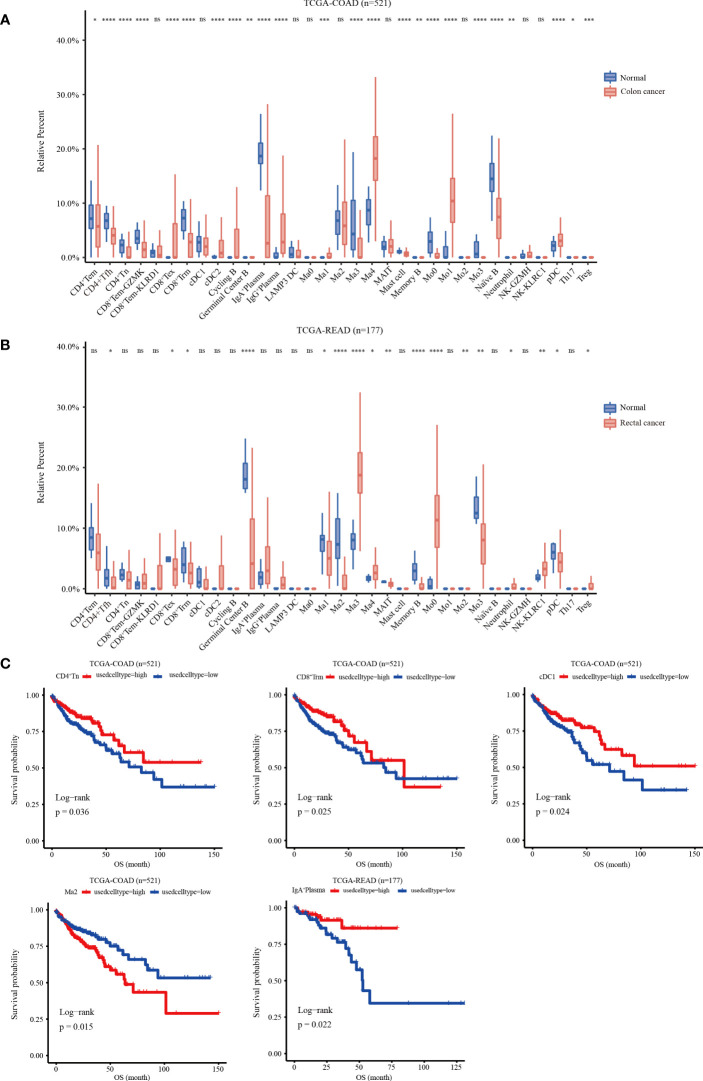
Relative infiltration abundance and prognostic significance of 33 immune cell subpopulations revealed by CIBERSORT deconvolution algorithm. **(A)** Relative infiltration abundance of 33 immune cell subpopulations identified by ScRNA-seq data in 480 colon cancer tissues and 41 adjacent tissues from the COAD cohort. **(B)** Relative infiltration abundance of 33 immune cell subpopulations identified by single-cell data in 167 colon cancer tissues and 10 adjacent tissues from the READ cohort, *p < 0.05, **p< 0.01, ***p< 0.001, ****p< 0.0001, and ns p > 0.05. **(C)** Kaplan-Meier overall survival curves of 460 patients in the TCGA-COAD cohort and 172 patients in the TCGA-READ cohort divided into the high infiltration group and low infiltration group, *p < 0.05.

A gene matrix was obtained from the scRNA-seq data to characterize the 33 immune cells. In addition, the bulk RNA-seq data from TCGA-COAD and READ cohorts were deconvoluted using CIBERSORT to calculate the relative abundance of each sample. The data showed that in the COAD cohort, the infiltration abundance of CD8^+^Tex, cDC2, IgG^+^PC, Ma1, Ma4, Mo1and pDC clusters in tumor tissues was higher than that in normal tissues. Conversely, higher infiltration abundance levels of CD4^+^Tem, CD4^+^Tfh, CD4^+^Tn, CD8^+^Tem-KLRD1, CD8^+^Trm, IgA^+^PCs, Ma3, mast cells, Mo0, Mo3, and Naïve B were found in normal samples (p < 0.05) (**Figure 5A**). In the READ cohort, the Ma3, Ma4, Mo0, neutrophil, NK-KLRC1, and Treg clusters showed higher infiltration abundance in tumor tissues; however, the expression levels of CD4^+^Tfh, CD8^+^Tex, CD8^+^Trm, germinal center B cells, Ma1, Ma2, MAIT, memory B, Mo3, and pDC clusters were higher in normal tissues than those in tumor tissues (p < 0.05) (**Figure 5B**). Next, we investigated the relationship between the infiltration abundance and overall survival (OS) with CRC. Our findings indicated that in the COAD cohort, the Ma2-APOE cluster was associated with a poor prognosis in colon cancer, whereas the cDC1, CD8^+^Trm, and CD4^+^Tn clusters were associated with a good prognosis. In the READ cohort, IgA^+^ plasma cell infiltration may predict a favorable prognosis for rectal cancer (**Figure 5C**)

### Identification of five TME subtypes characterized by immune cells deconvolution in CRC and their prognostic significance

**Figure 6:**
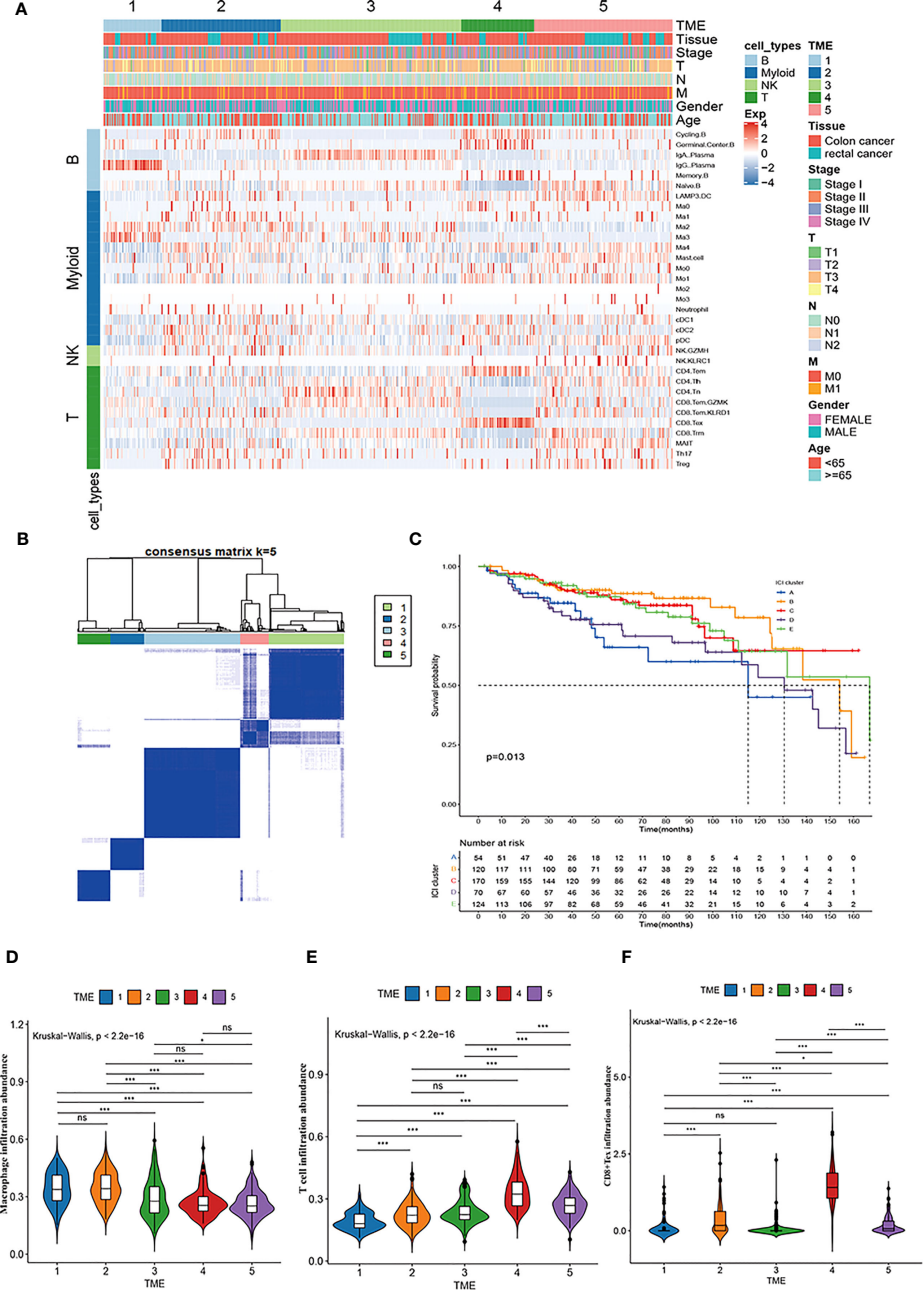
Immune cell characteristics and prognostic significance of TME subtypes in CRC. **(A)** Heatmap showing unsupervised clustering of 5 TME subtypes of immune patterns in the TCGA cohort, with the rows representing the 33 immune subpopulations identified by the ScRNA-seq data set, and the columns representing 647 CRC patients from the TCGA-COAD and READ cohorts; hierarchical clustering according to TME subtype, histological site, disease stage, tumor-node-metastasis (TNM) stage, and age. **(B)** Consensus matrix heatmap representing the consensus matrix with k=5 by consensus clustering; the range of value from 0 to 1 implying the probability in the same cluster with the color scaling from white to dark blue. **(C)** Kaplan-Meier overall survival curves of 5 TME subtypes in TCGA-COAD and READ cohorts. **(D-F)** Violin plot showing the representative immune cell abundance of 5 TME subtypes, including macrophages **(D)**, T cells **(E)**, and CD8^+^ Tex cells **(F)**, compared by Kruskal-Wallis test.

We used the CIBERSORT deconvolution algorithm to infer the composition of 33 of the immune cell subtypes in the bulk RNA sequence data from the TCGA-COAD and READ cohorts. A total of 623 CRC patients from TCGA cohorts were clustered into five different TME subtypes (TME 1-5) by consensus clustering method and the relationship between the different subtypes and clinical characteristics (including: age, sex, TNM, stage and tissue location) was illustrated by heatmap (**Figures 6A, B**). Further, the overall survival of CRC patients from TCGA cohort was assessed by Kaplan-Meier survival analysis, which confirmed significant differences in the prognosis of CRC patients with the five TME subtypes. Notably, the TME-1 subtype represented a significantly reduced proportion of T-cell infiltration and the highest proportion of macrophages, which had the worst prognosis (**Figures 6C-E**). Although the TME-4 subtype had the highest proportion of T cell infiltration, it mainly showed CD8^+^Tex subtype, lacking infiltration of cytotoxic T cells, and therefore had a poor prognosis (**Figures 6A, C, E, F**).

### CellChat analysis of immune cell communication in CRC

**Figure 7:**
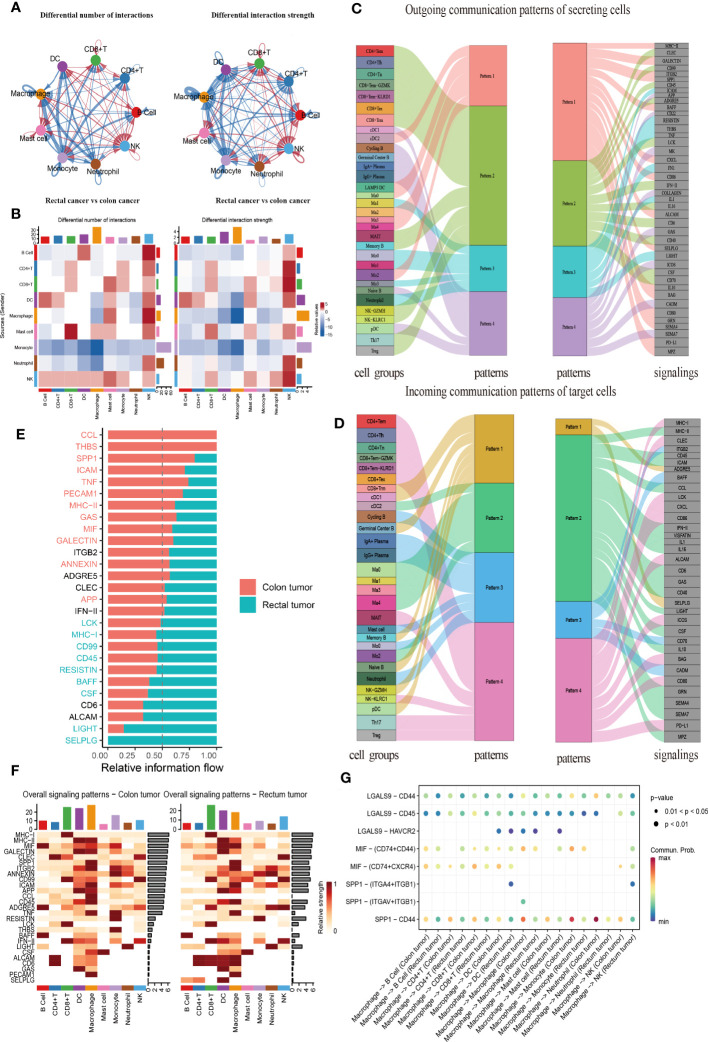
CellChat analysis of the crosstalk between immune cells in colon cancer or rectal cancer. **(A)** Comparisons of overall changes in cell-cell communication between rectal cancer and colon cancer, including the differential number of interactions (left) and differential interaction strength (right) between immune cells of rectal cancer compared with colon cancer, with the blue line representing reduced communication in rectal cancer compared to colon cancer, while the red line representing increased communication in rectal cancer compared to colon cancer. **(B)** Heatmaps showing the interaction number (left) and interaction strength (right) between colon cancer and rectal cancer, with the top color bar representing the sum of the column values displayed in incoming signals and the right color bar representing the sum of outgoing signals, red or blue indicating increased or decreased signal of colon cancer compared with normal control. **(C)** Outgoing signal pattern of immune cells acting as secretory cells, and the pattern corresponding to signaling pathways. **(D)** Incoming signal pattern of immune cells acting as target cells, and the pattern corresponding to signaling pathways; the thickness of the flow indicating the contribution to each pattern. **(E)** Differences in the overall signaling pathway between colon cancer and rectal cancer, with the ranking indicating the importance of the pathways; red indicating the signaling pathways enriched in colon cancer, green representing the signaling pathways enriched in rectal cancer, and black representing no difference in signaling pathway enrichment in colon cancer and rectal cancer. **(F)** Heatmaps of the overall signaling pathway of each immune cell subpopulation mediated by individual signaling pathway in colon cancer (left) and rectal cancer (right). **(G)** Communication probabilities of important ligand-receptor pairs from macrophages to individual immune cells in colon and rectal cancers, with the dot color reflecting the communication probability, blank indicating the communication probability zero, and dot size representing the p value.

CellChat was used to comprehensively assess immune cell interactions between colon or rectal cancer tissues and normal tissues in terms of the number and weight of cell communications (**Figure 7A**, **Supplementary Figures 4A, B**). In terms of incoming signals, the interaction number of macrophages increased significantly, the interaction weight of DC signals was elevated most substantially, and the interaction number and weight of monocytes decreased most significantly in colon cancer tissues when compared with normal tissues. Regarding outgoing signals, the number and weight of communication between monocytes and DCs were prominently increased, whereas the number and weight of communication between monocytes and neutrophils were most significantly reduced (**Supplementary Figure 4C**). Compared with those in normal tissues, the number of incoming signals of macrophages and DCs increased, but the weight of macrophages decreased and the number and weight of monocytes decreased most significantly in rectal cancer tissues. For the outgoing signals, macrophages and DCs both showed increases in interaction quantity and weight, while monocytes and neutrophils both had significantly decreased interaction number and weight (**Supplementary Figure 4D**). Further, the difference in the interaction between colon and rectal cancer tissues was analyzed, the results of which suggested elevated incoming signals from NK cells but diminished incoming signals from macrophages and outgoing signals from monocytes in rectal cancer tissues as compared to colon cancer tissues (**Figure 7B**). In conclusion, the interaction between myeloid cells and other cells was significantly changed in both the comparison between normal tissues and cancer tissues and the comparison between colon cancer and rectal cancer.

Importantly, CellChat further uncovered the patterns of incoming and outgoing signals. At the outgoing end of the signaling pathway, immune cell subsets acted as secretory cells to send signals principally through four patterns. Specifically, T and NK cells drove CD99, CD45, and ADGRE5, as well as INF-II and interleukin signaling pathways, mainly through Pattern 2. The major B cell subsets cDC1 and pDC mediated CD22, GAS, and ICOS signaling pathways primarily through Pattern 4. Myeloid cell clusters drove MHC II, SPP1, BAFF, CXCL, CD86, and PD-L1 signaling pathways through Pattern 1. Additionally, Ma1, Mo0, Mo3, and neutrophils jointly promoted ICAM, TNF, FN1, and other pathways through Pattern 3 (**Figure 7C**). More importantly, T, B, and myeloid cells were dominated by Pattern 4, Pattern 3, and Pattern 2, respectively, when immune cell subsets served as the targeted cells at the incoming end of the signaling pathway. Pattern 1 corresponded to the incoming signals from numerous immune cells, which were mainly driven by ADGRE5 and SELPLG signaling pathways (**Figure 7D**).

All communication probabilities in the information network were summarized to compare the difference in overall information flow between colon and rectal cancers. The results unraveled that CCL5, THBS, SPP1, ICAM, and TNF signaling pathways were more abundant in colon cancer (red), whereas SELPLG, LIGHT, CSF, and BAFF signaling pathways were more abundant in rectal cancer (green) (**Figure 7E**). The visualization results of heat maps revealed an elevation in the overall information flow from CD8^+^ T cell, DC, and macrophage clusters in both colon and rectal cancer tissues, the most significant increase in the information flow from macrophages in colon cancer tissues, and a dominant increase in the information flow from CD8^+^ T cells in rectal cancer tissues (**Figure 7F**).

As macrophages are key to cell communication in CRC and are heterogeneous in communication across tissues, the probability of ligand-receptor communication between macrophages and other immune cells was further compared between colon and rectal cancer tissues. The results depicted that macrophages were critical for regulating cell-cell communication in CRC and that tissue variation existed in communication patterns. SPP1-CD44 (L-R) was highly active in the communication between macrophages and other cells and more active in colon cancer tissues than in rectal cancer tissues throughout intercellular information interaction as it mediated the immunosuppression and progression of CRC. Because the ligand MIF is a chemokine-like inflammatory mediator, its multi-subunit receptor complexes CD74-CXCR4 and CD74-CD44 can orchestrate inflammatory pathways. Our findings manifested that CD74-CXCR4 and CD74-CD44 were highly activated in the signal flow from macrophages to B and T lymphocytes (**Figure 7G**). The Ma1 subgroup exerted the strongest effect on these receptor-ligand pairs (**Supplementary Figure 4E**). Therefore, this result supported our previous inference that Ma1 was a kind of M2-like TAMs.

### Multiplex immunohistochemistry description the interaction between SPP1^+^TAM and Treg in the TME of CRC

**Figure 8:**
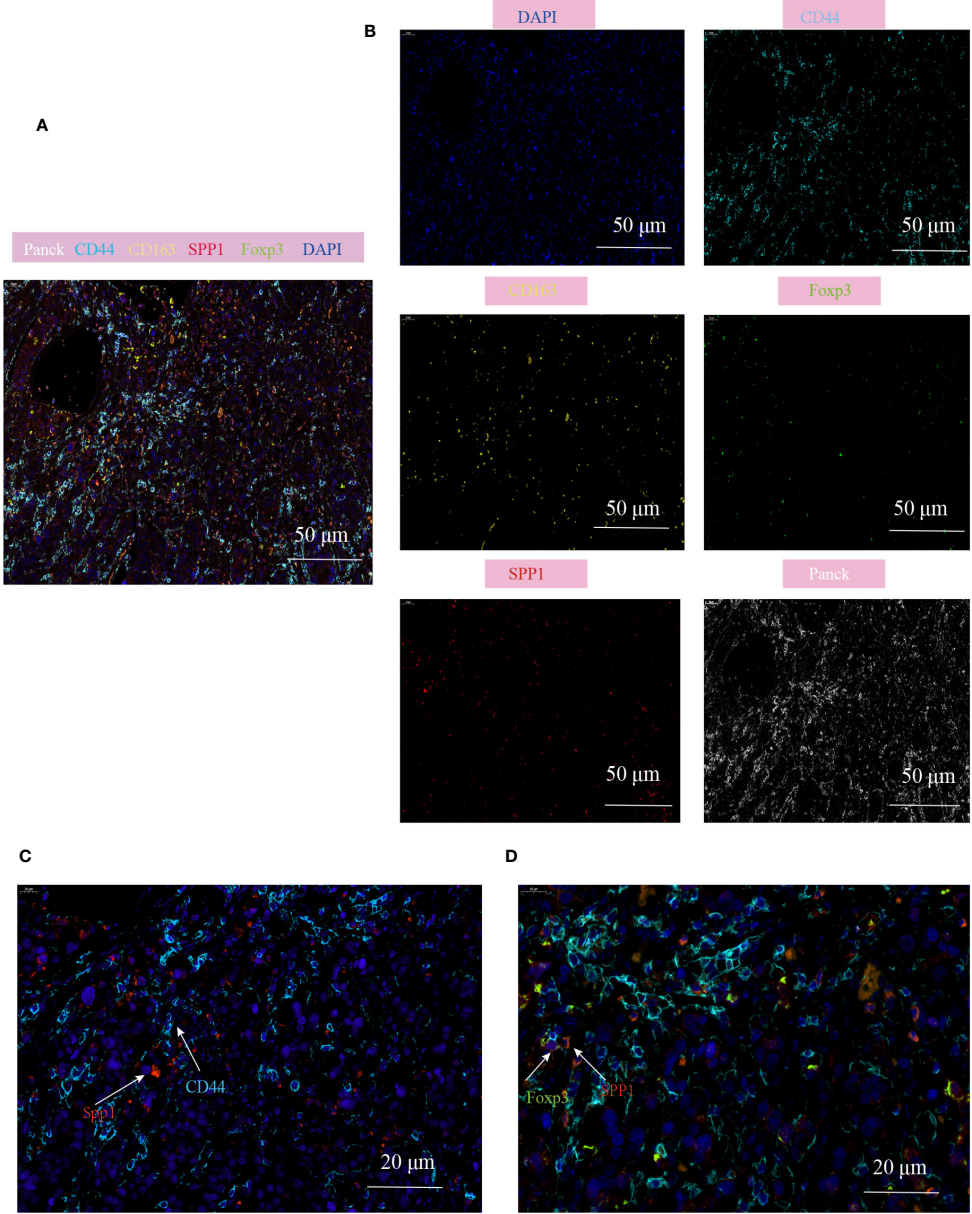
Multiplex immunofluorescence showing the interaction between SPP1^+^TAM and Foxp3^+^Treg in the TME of CRC. **(A, B)** Multiplex immunofluorescence images demonstrating the localization of different cell populations in CRC, using typical marker genes including Panck (white), CD44 (cyan), CD163 (yellow), SPP1(red), Foxp3 (green), DAPI (blue), scale bar=50µm; **(C)** Representative images of SPP1-CD44 mediated co-localization of cell populations in CRC patients, scale bar=20µm; **(D)** Representative images of interaction of SPP1^+^TAM and Foxp3^+^Treg cells in the CD44 enriched regions, scale bar=20µm.

To validate the cell communication results based on Cellchat analysis, mIHC technology was used to verify the cell population interactions mediated by the high active L-R interaction. The previous prediction of L-R interactions found that SPP1-CD44 interation revealed strong effects on the interaction between macrophages and Treg subgroups (**Supplementary Figure 4E**), so we analyzed the co-localization of SPP1^+^TAM and Treg in the TME of CRC. Consistent with our prediction, the prevalence of SPP1^+^TAMs co-localizing with Foxp3^+^Tregs and the proximity of their spatial locations within the CD44 enriched regions, led us to hypothesize that the crosstalk between SPP1^+^TAMs and Foxp3^+^Tregs increases the immunosuppressive effect, which is most likely mediated by SPP1-CD44 (**Figures 8A-D**, **Supplementary Figures 5A, B**).

## Discussion

The inter- and intratumoral heterogeneities of immune cell tumors directly affect the prognosis of patients and their response to immunotherapy. In this study, two CRC 10xGenomics scRNA-seq datasets were integrated, including 33 patients and 6,2398 immune cells re-clustered into 33 immune cell clusters, to characterize the immune cell landscape of CRC and comprehensively analyze the phenotypic and molecular differences and intercellular communication between immune cells in CRC at single-cell resolution. In addition, the heterogeneity of colon cancer, rectal cancer, and normal adjacent tissues in the immune microenvironment and their differences in cell-interaction patterns were compared. Furthermore, we combined bulk RNA-seq data from TCGA cohorts to evaluate the prognostic value of these pivotal immune cell subpopulations. In addition, according to the characteristics of immune infiltration, patients with CRC were divided into five TME subtypes with different prognostic characteristics.

TAMs are the most important myeloid cells in the immunosuppressive microenvironment of tumors (38, 55). In the present study, the diversity and complexity of myeloid cells were investigated. Five macrophage phenotypes and four different subtypes of DCs were identified. More importantly, we observed a heterogeneous distribution of myeloid cells in CRC and normal adjacent tissues. We found an important Ma1-SPP1 macrophage that exhibited M2-like phenotypes, which potentially promoted angiogenesis and increased infiltration abundance in tumor tissues. Therefore, we speculate that Ma1-SPP1 may be an important TAM. Interestingly, however, when we compared the inter-tissue heterogeneity based on our analysis of the single-cell dataset, the proportion of Ma1-SPP1 macrophages in colon cancer tissue was higher than that in normal tissue, whereas the increase in rectal cancer was not statistically significant. In addition, we did not observe significant differences in the abundance of infiltrating macrophage subsets between colon and rectal cancers.

Importantly, we found that myeloid cells play an important role in cell–cell communication. In different pathological tissues, myeloid cells show differences in the strength of their interaction and signaling pathways with other cells. This difference may have a more significant impact on the tumor microenvironment than on the abundance of infiltration. Specifically, the interaction of macrophages with DCs and other immune clusters increased in CRC tissues, whereas monocyte communication decreased. Compared to colon cancer, the communication signals of macrophages and monocytes are decreased in the TME of patients with rectal cancer. Among these, the SPP1 signaling pathway, a characteristic gene of the Ma1 subgroup, is highly active in colon cancer, whereas the SELPLG and LIGHT signaling pathways are highly active in rectal cancer. Interestingly, we observed a high probability of SPP1-CD44 mediated information flow in the cell–cell communication between Ma1 macrophages and Tregs. We hypothesized that SPP1-CD44 information flow mediates intercellular crosstalk between TAMs and Tregs, which enhances the immunosuppressive microenvironment of CRC. Furthermore, through mIHC, we confirmed the spatial co-localization of SPP1^+^TAMs and Tregs, and this cell–cell interaction was more prominent in CD44 enriched areas. Although we were able to identify SPP1 as a ligand of TAMs that interacts with CD44 high-expressing cells, we could not confirm whether CD44 was directly involved in intercellular crosstalk as a receptor for Tregs or whether other CD44^+^cell populations acted as a bridge to increase Treg infiltration due to the low cell specificity of CD44.

cDC1s are the main APC cells responsible for antigen cross-presentation, and the priming of CD8^+^T cells is crucial for antitumor responses (26). Recruitment and expansion of cDC1 in the TME were associated with increased CD8^+^T cell infiltration and a good prognosis and exhibited a better clinical response to ICI. A study of Notch-regulated dendritic cells inhibiting the development of inflammation-associated CRC revealed a direct relationship between Notch2 signaling and infiltrating cDC1s as well as an association between the inhibition of cDC1 signaling and poor prognosis in human CRC. The study indicated that decreased intratumoral cDC1s and circulating cDC1s in patients with CRC are related to disease stage, whereas suppressed cDC1 gene signature expression in human CRC is associated with a poor prognosis (56). In addition, another study found that colorectal tumors can be further sensitized to immune checkpoint therapy using a combination of low-dose chemotherapy and oncolytic HSV-1 in a mouse model of dMMR CRC, mainly through the mechanism of making tumors sensitive to immunotherapy by promoting high levels of cDC1 infiltration in tumors after treatment, and the therapeutic effect depends on the presence of cDC1s (57). Our study observed a significant reduction in the abundance of cDC1 infiltration in colon cancer in the scRNA-seq cohort, and high infiltration of cDC1 was found to be correlated with good outcomes in TCGA-COAD cohort. Therefore, our findings support cDC1 as a potential biomarker for predicting OS in patients with CRC.

The enrichment of PCs in tumors significantly correlates with the aggregation of tertiary lymphoid structures (TLSs) (58). PCs produced in situ in tumor TLSs can generate antibodies against specific tumor-related antigens, which exert anti-tumor or tumor-promoting effects in different TMEs (59, 60). The enrichment of PCs in some tumors also serves as a prognostic indicator for PD-L1 inhibitor therapy (31, 61). After reclustering the single-cell data of CRC, we identified PCs with IgA^+^ PC and IgG^+^ PC phenotypes, presenting a significantly heterogeneous distribution in the tumor and normal tissues. Specifically, IgA^+^ PCs are decreased in colorectal tumor tissues, whereas IgG^+^ PCs are enriched in tumor cells. IgA ^+^ PCs are usually believed to be abundantly produced by the intestinal mucosa and can migrate to target tissues (62). Therefore, IgA^+^ PCs have been detected in the microenvironments of multiple tumors. However, the role of IgA^+^ PCs in tumor development and progression has not been unanimously determined (63, 64). In CRC, IgA^+^ PCs inhibit the activation of cytotoxic CD8^+^ T cells, leading to a poor prognosis (54). In contrast, another study reported that IgA^+^ PCs are significantly associated with the long-term survival of patients with rectal cancer (65). By analyzing the clinical data of colon adenocarcinoma (COAD) and rectum adenocarcinoma (READ) from the Cancer Genome Atlas (TCGA) database, we found that the infiltration of IgA^+^ PCs was associated with a prolonged OS of patients with rectal cancer. Hence, the enrichment of IgA^+^ PCs may contribute to the prognosis of rectal cancer; however, no correlation was observed between the enrichment of IgA ^+^ PCs and the prognosis of colon cancer. Accumulating evidence suggests that IgG antibodies produced by IgG^+^PCs can enhance the T cell response (66). In addition, IgG antigens can directly induce antibody-dependent cellular cytotoxicity (ADCC) via Fc receptor activation (67). Nevertheless, in complement-rich tumors, IgG antibodies activate the complement cascade, thus producing anaphylatoxins and promoting inflammation and angiogenesis (32). We found that IgG^+^ PCs were enriched in colon cancer tissues; however, whether IgG^+^ PC enrichment was indicative of a better prognosis was not determined. However, the role of IgG^+^ PCs in the TME requires further experimental verification.

CD103 is recognized as a marker of Trm cells, and it is generally believed that Trm cells express high levels of PD-1, TIGIT, and CD39 (68). The co-expression of CD103 and CD39 has been confirmed to be a marker for the identification of tumor-reactive CD8^+^TIL in human solid cancers (20, 69). Notably, Duhen et al. confirmed that the percentage of CD103^+^CD39^+^CD8^+^ TILs was high in MSI-high colon cancer with high mutational burden, which showed the highest response rates to immunotherapy. In contrast, the percentage of CD103^+^CD39^+^CD8^+^ TILs was low in patients with microsatellite-stable colon cancer and colorectal liver metastasis, who tended to respond poorly to immunotherapy (69). Some studies have suggested that triple-positive TIL exhibit a strong activation/exhaustion phenotype and have a superior prognostic impact compared to TIL expressing other combinations of these markers (20). Interestingly, we found that the abundance of PD1^+^CD39^+^CD103^+^TILs was extremely low in CRC, whereas a few triple-positive cells were mainly distributed in the Th17 and CD8^+^Tem-KLRD1 subpopulations. Double-positive cells accounted for a high proportion, and CD8^+^Trm was the predominant subpopulation of CD39^+^CD103^+^T cells. The expression of PD-1 was not upregulated in CD8^+^Trm; however, the expression of HAVCR2 was significantly upregulated. Highly infiltrated CD8^+^Trms were associated with prolonged OS in CRC but not with the prognosis of rectal cancer. Therefore, we believe that CD39^+^CD101^+^CD8^+^Trm could better predict the tumor reactivity of CD8^+^TIL in colon cancer.

In conclusion, this study comprehensively analyzed the immune cell atlas of human CRC at the single-

cell level. Specifically, the heterogeneity distribution and phenotype of immune cells were deeply analyzed in colon cancer and rectal cancer, followed by the characterization of the pathway enrichment, cell communication, and transcription factors of each immune cell subset. Further, the prognostic role of major TILs and TME subtypes in CRC was evaluated by integrating bulk RNA transcriptome data. These findings provide novel insight into the immunotherapy of CRC.

## Data availability statement

The datasets presented in this study can be found in online repositories. The names of the repository/repositories and accession number(s) can be found within the article/**Supplementary Material**.

## Ethics statement

The studies involving human participants were reviewed and approved by the ethics committee of General Hospital of Northern Theater Command. The patients/participants provided their written informed consent to participate in this study.

## Author contributions

QZ conceived and designed the study. YL performed single cell bioinformatics analysis, and YL, QZ processed statistical analysis. CZ, XW helped provide clinical sample collection and histopathological experiments. MH and YL helped review the paper and provided key data explanations. ZQ wrote the manuscript according to the opinions of all the authors. All authors have read and approved the manuscript.
