# Supplementary material for: Integration of single-cell RNA sequencing and bulk RNA transcriptome sequencing reveals a heterogeneous immune landscape and pivotal cell subpopulations associated with colorectal cancer prognosis
Source: Front Immunol. 2023 Aug 22;14:1184167. doi: 10.3389/fimmu.2023.1184167 (PMC10477986; doi:10.3389/fimmu.2023.1184167)
Supplement: Supplementary file 1 [file DataSheet_1.docx]

# Supplementary Figures


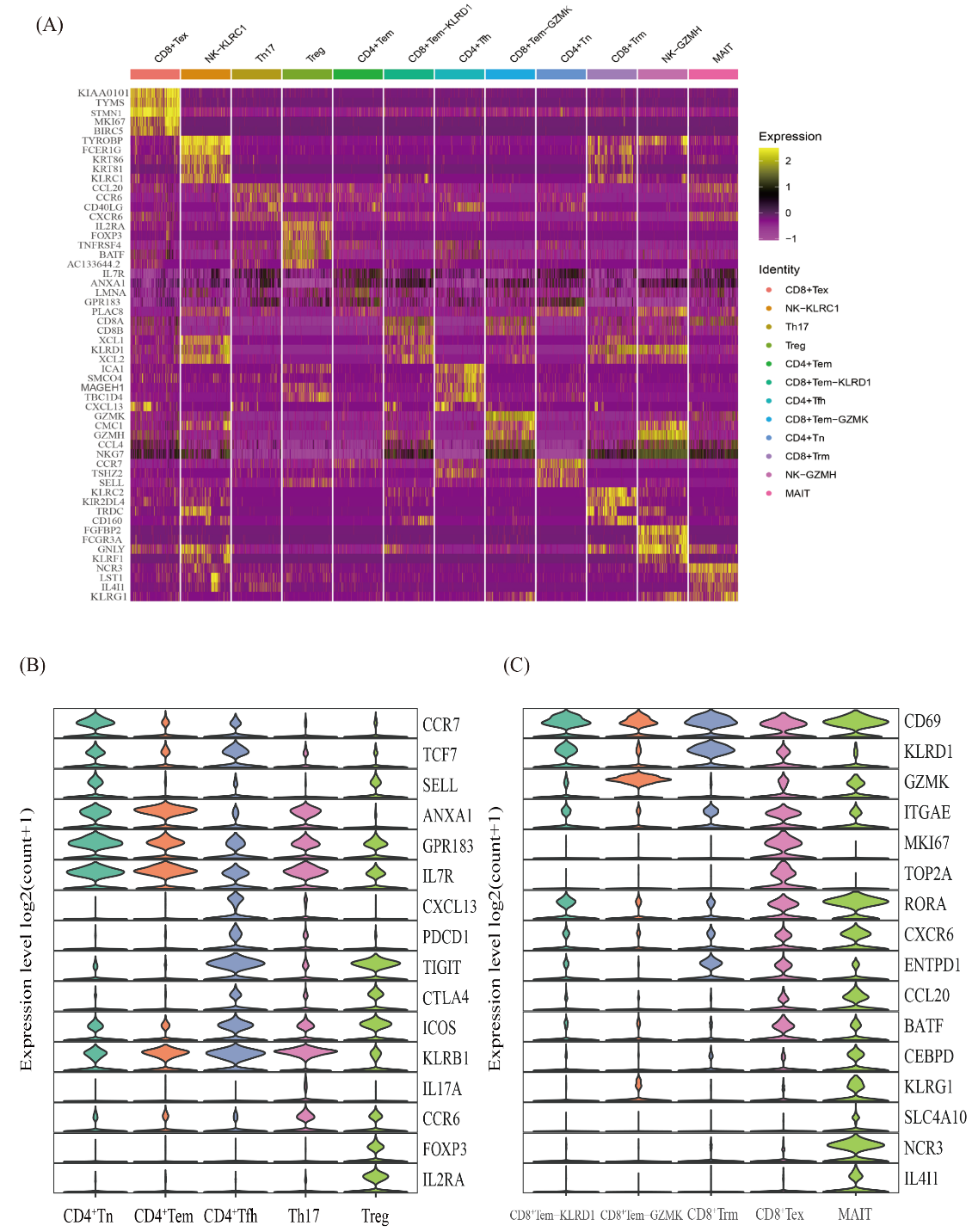


**Supplementary Figure 1.** (A). Heatmap showing the TOP 5 DEGs of each subgroup of T & NK. (B). Violin plot of relative expression of key characteristic genes in CD4+T clusters. (C). Violin plot of relative expression of key characteristic genes in CD8+T clusters. The relative expression of genes was calculated by log (count +1).


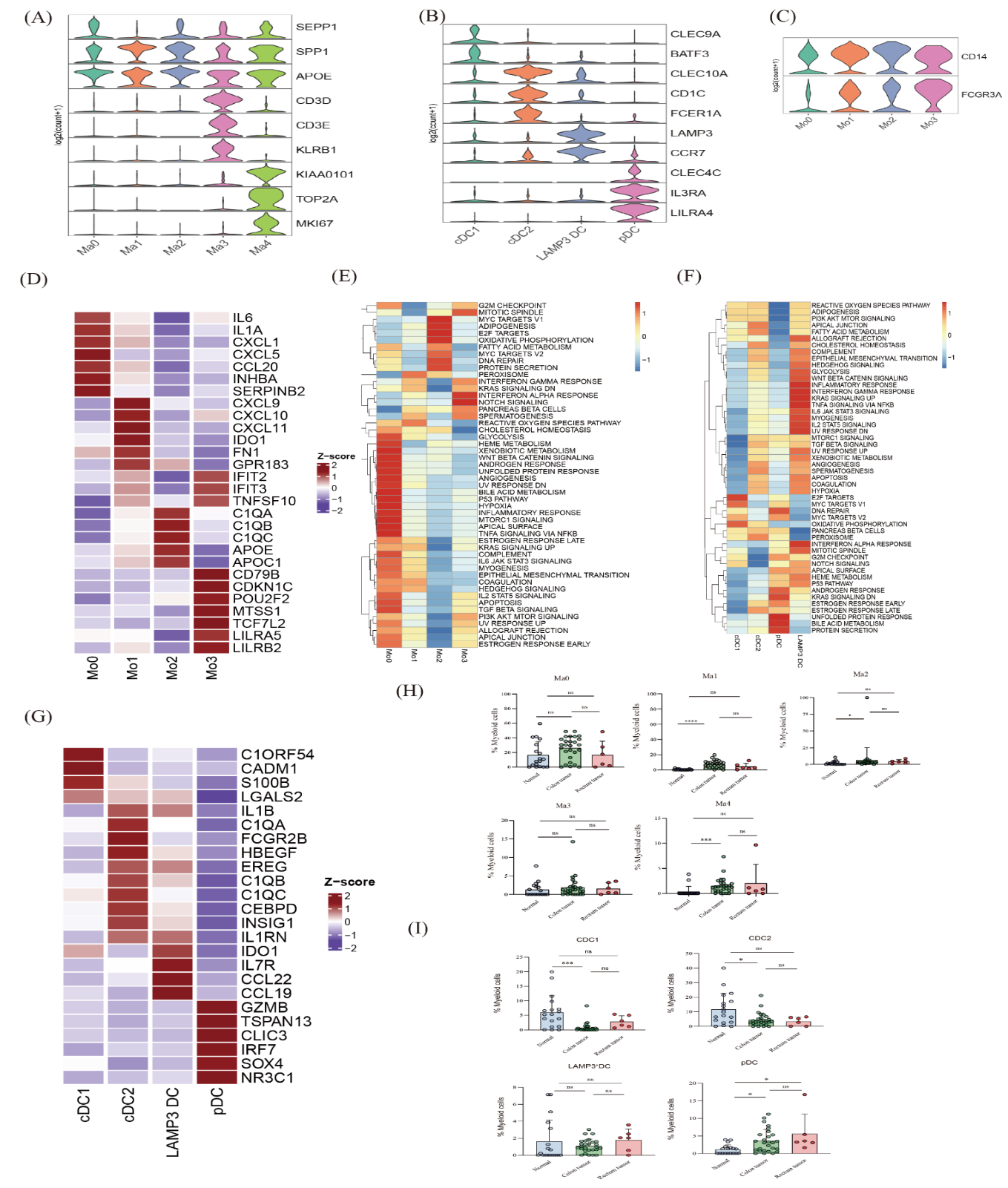


**Supplementary Figure 2. (A)** Violin plot showing the log2(count+1) expression of major marker genes in each macrophage cluster. **(B)** Violin plot showing the log2(count+1) expression of major marker genes in each DC cluster. **(C)**The relative expressions of CD14 and FCGR3A (CD16) were calculated as log (count+1). **(E)** Heatmap showing the enriched pathways from hallmark gene sets in monocytes clusters using gene set variation analysis (GSVA). **(F)** Heatmap showing the enriched pathways from hallmark gene sets in DC clusters using gene set variation analysis (GSVA). **(G)** Heatmap showing the key differentially expressed genes (DEGs) of each DC cluster. **(H)** Box plots illustrating the relative fractions of the 5 macrophage subtypes in myeloid lineage in different samples**,** * p < 0.05, ** p < 0.01, **** p < 0.0001, ns p > 0.05. **(I)** box plots illustrating the relative fractions of the 4 DC subtypes in myeloid lineage in different samples, * p < 0.05, *** p < 0.001, ns p > 0.05.


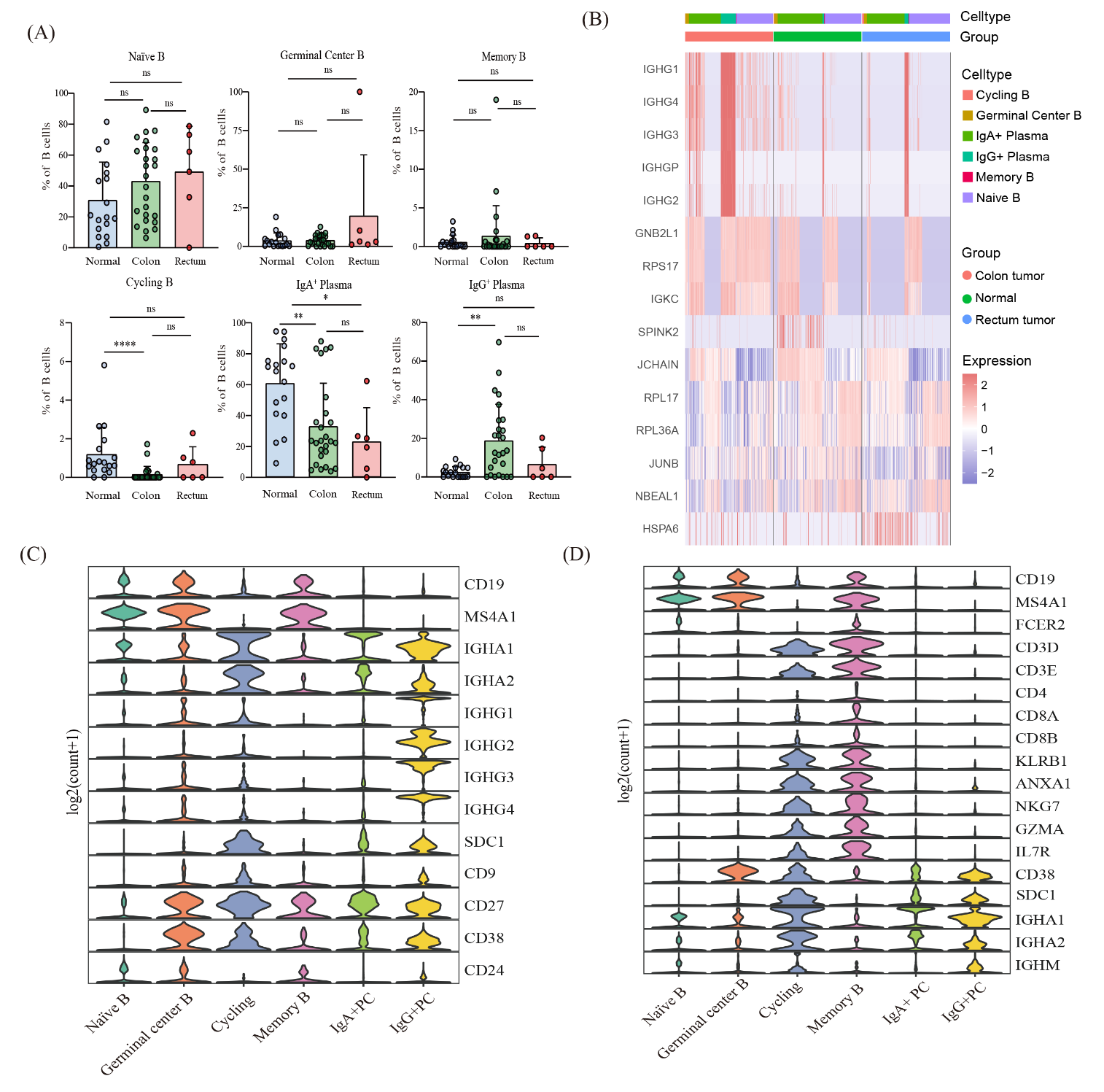


**Supplementary Figure 3. (A)** Proportion of each B cell subtype accounting for the total number of immune cells in colon cancer, rectal cancer, and adjacent normal tissues. **(B)**Heatmap showing the top DEGs in B subtypes between colon cancer, rectal cancer and normal. (**C), (D)** Violin plot of relative expression of key genes in B lineage clusters.


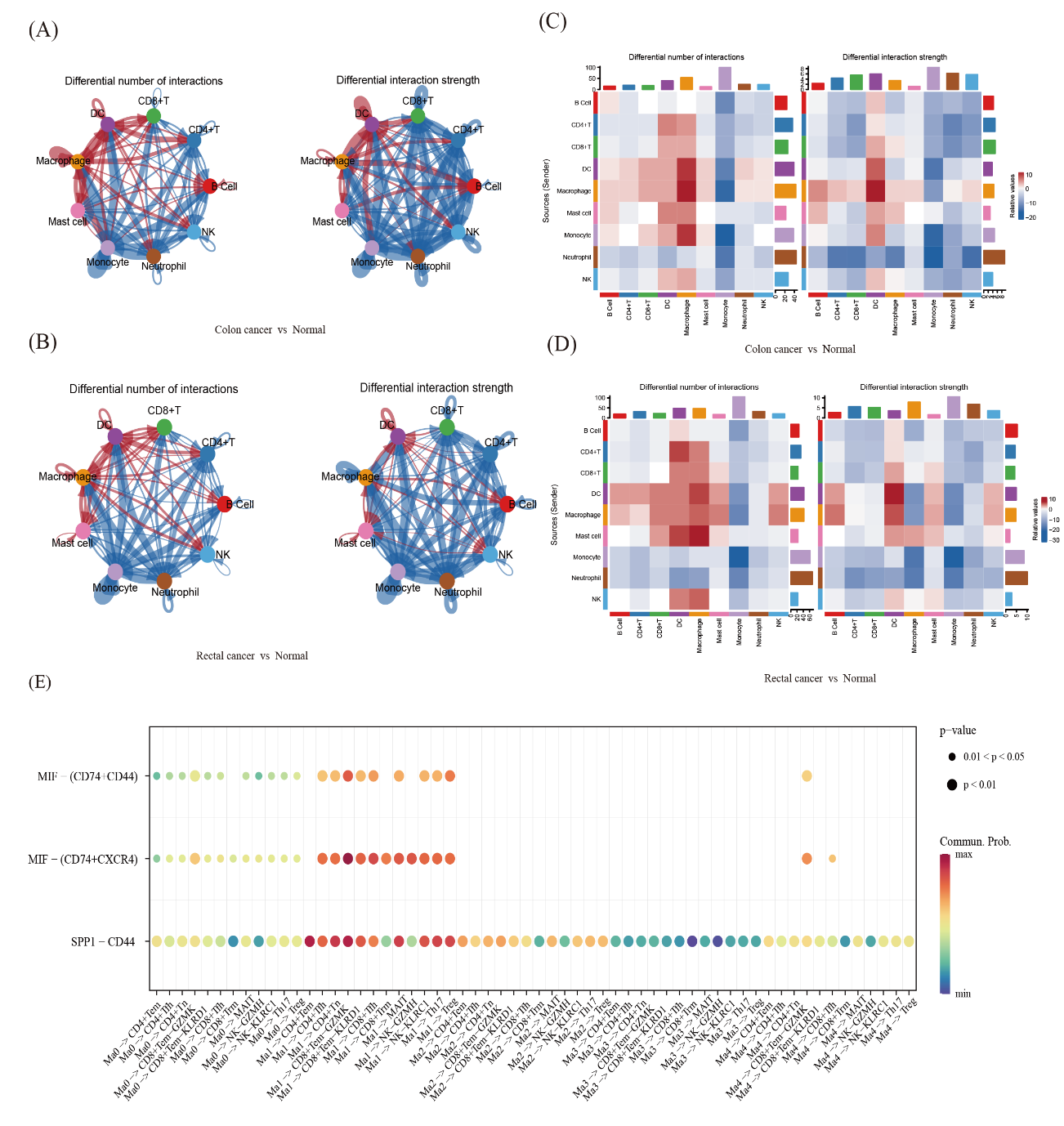


**Supplementary Figure 4. (A)** The overall number and interaction strength changes in cell-cell communication between colon cancer and normal. **(B)**The overall number and interaction strength changes in cell-cell communication between rectal cancer and normal. **(C)** Heatmaps of the interaction number (left) and interaction strength (right) between colon cancer and normal. **(D)** Heatmaps of the interaction number (left) and interaction strength (right) between rectal cancer and normal. **(E)** Communication probabilities of SPP1-CD44, MIF- (CD74+CD44) and MIF- (CD74+CXCR4) between macrophage subpopulations and T &NK subpopulations.


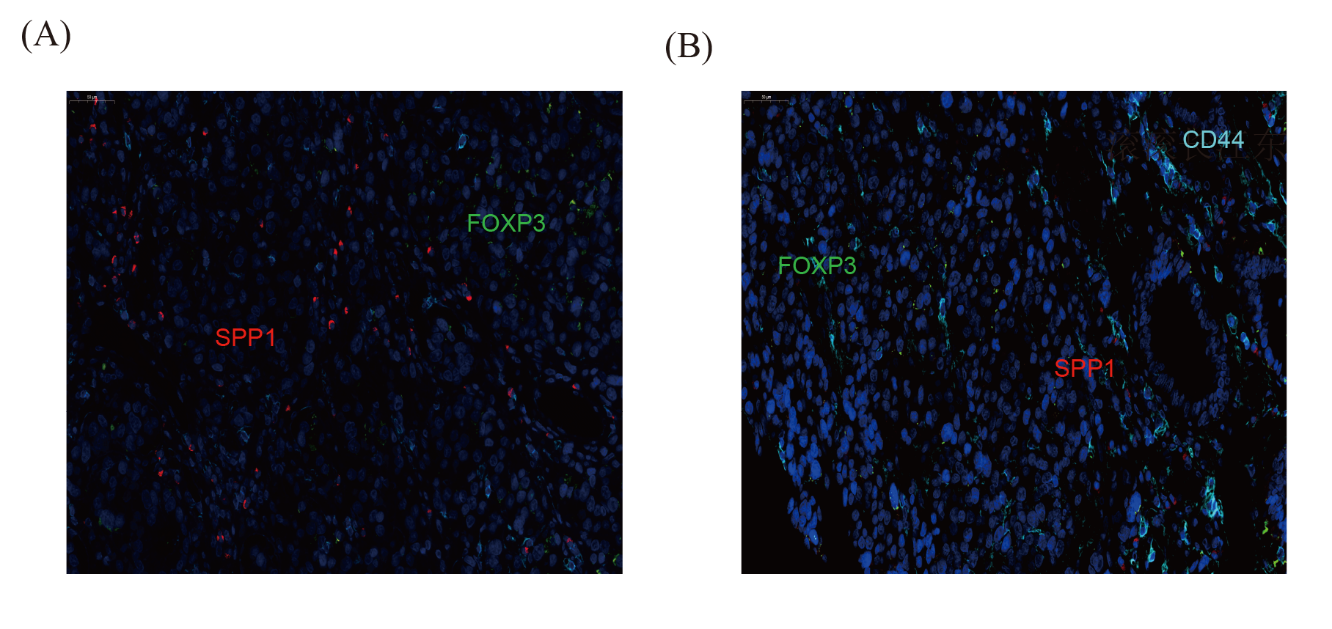


**Supplementary Figure 5. (A)** The main expression and distribution of SPP1^+^TAM in CD44 deficient region, and the spatial location with scattered FOXP3^+^Treg. **(B)** The main expression and distribution of FOXP3^+^Treg in CD44 deficient region, and the spatial location with scattered SPP1^+^TAM.
