# Supplementary material for: Integration of single-cell RNA sequencing and bulk RNA transcriptome sequencing reveals a heterogeneous immune landscape and pivotal cell subpopulations associated with colorectal cancer prognosis
Source: Front Immunol. 2023 Aug 22;14:1184167. doi: 10.3389/fimmu.2023.1184167 (PMC10477986; doi:10.3389/fimmu.2023.1184167)
Supplement: Supplementary file 2 [file Table_1.docx]

Table S1. Clinical Patient Information

| Patient | Normal | Tumor | Histological type | Gender | Age | T | N | M | Stage |
| --- | --- | --- | --- | --- | --- | --- | --- | --- | --- |
| C663 | C663-TP | C663-TC | Colon cancer | M | 31 | T4 | N1 | M0 | IIIB |
| C679 | C679-TP | C679-TC | Colon cancer | F | 48 | T4 | N1 | M0 | IIIB |
| C684 | C684-TP | C684-TC | Colon cancer | F | 63 | T4 | N2 | M0 | IIIC |
| C694 | C694-TP | C694-TC | Colon cancer | M | 58 | T4 | N1 | M0 | IIIB |
| C697 | C697-TP | C697-TC | Colon cancer | F | 58 | T4 | N2 | M0 | IIIC |
| C669 | C669-TP | C669-TC | Rectal cancer | M | 67 | T4 | N2 | M0 | IIIC |
| C688 | C688-TP | C688-TC | Rectal cancer | M | 65 | T4 | N1 | M0 | IIIB |
| C691 | C691-TP | C691-TC | Rectal cancer | M | 40 | T4 | N1 | M0 | IIIB |
| C698 | C698-TP | C698-TC | Rectal cancer | F | 66 | T4 | N2 | M0 | IIIC |
