# Supplementary material for: Integration of single-cell RNA sequencing and bulk RNA transcriptome sequencing reveals a heterogeneous immune landscape and pivotal cell subpopulations associated with colorectal cancer prognosis
Source: Front Immunol. 2023 Aug 22;14:1184167. doi: 10.3389/fimmu.2023.1184167 (PMC10477986; doi:10.3389/fimmu.2023.1184167)
Supplement: Supplementary file 3 [file Table_2.docx]

| **Table 2 Patient information in GSE132465 and GSE1467771 datasets** | | | | | | | | | | | | |
| --- | --- | --- | --- | --- | --- | --- | --- | --- | --- | --- | --- | --- |
| **Platform** |  | **Patient** | **Normal** | **Tumor** | **Histological type** | **Gender** | **Age** | **T** | **N** | **M** | **Stage** | **MSI** |
| 10x Genomics | **GSE132465** | SMC01 | SMC01-N | SMC01-T | **Rectum cancer** | F | 64 | 3 | 0 | 0 | IIA | MSS |
|  |  | SMC02 | SMC02-N | SMC02-T | **Rectum cancer** | M | 66 | 3 | 1b | 0 | IIIB | MSS |
|  |  | SMC03 | SMC03-N | SMC03-T | Colon cancer | F | 83 | 4b | 2a | 0 | IIIC | MSI-H |
|  |  | SMC04 | SMC04-N | SMC04-T | Colon cancer | M | 69 | 3 | 1b | 0 | IIIB | MSS |
|  |  | SMC05 | SMC05-N | SMC05-T | Colon cancer | F | 58 | 3 | 0 | 0 | IIA | MSS |
|  |  | SMC06 | SMC06-N | SMC06-T | Colon cancer | M | 46 | 3 | 1b | 0 | IIIB | MSI-H |
|  |  | SMC07 | SMC07-N | SMC07-T | Colon cancer | F | 67 | 2 | 0 | 0 | I | MSS |
|  |  | SMC08 | SMC08-N | SMC08-T | Colon cancer | M | 68 | 3 | 1b | 0 | IIIB | MSS |
|  |  | SMC09 | SMC09-N | SMC09-T | Colon cancer | M | 75 | 3 | 0 | 0 | IIA | MSS |
|  |  | SMC10 | SMC10-N | SMC10-T | Colon cancer | F | 77 | 3 | 0 | 0 | IIA | MSI-H |
|  |  | SMC11 |  | SMC11-T | Colon cancer | F | 38 | 2 | 1a | 0 | IIIA | MSS |
|  |  | SMC14 |  | SMC14-T | Colon cancer | M | 77 | 4a | 1b | 0 | IIIB | MSS |
|  |  | SMC15 |  | SMC15-T | Colon cancer | M | 56 | 3 | 0 | 0 | IIA | MSS |
|  |  | SMC16 |  | SMC16-T | Colon cancer | M | 59 | 4a | 1b | 0 | IIIB | MSS |
|  |  | SMC17 |  | SMC17-T | Colon cancer | M | 47 | 4a | 1b | 0 | IIIB | MSS |
|  |  | SMC18 |  | SMC18-T | Colon cancer | F | 63 | 3 | 0 | 0 | IIA | MSS |
|  |  | SMC19 |  | SMC19-T | Colon cancer | F | 80 | 3 | 1 | 0 | IIIB | MSS |
|  |  | SMC20 |  | SMC20-T | Colon cancer | F | 65 | 4a | 1c | 0 | IIIB | MSS |
|  |  | SMC21 |  | SMC21-T | **Rectal cancer** | M | 51 | 3 | 1 | 1a | IVA | MSS |
|  |  | SMC22 |  | SMC22-T | Colon cancer | M | 76 | 3 | 1b | 0 | IIIB | MSS |
|  |  | SMC23 |  | SMC23-T | Colon cancer | F | 67 | 3 | 1b | 0 | IIIB | MSS |
|  |  | SMC24 |  | SMC24-T | Colon cancer | F | 48 | 1 | 0 | 0 | I | MSI-H |
|  |  | SMC25 |  | SMC25-T | Colon cancer | F | 57 | 3 | 2b | 1a | IVA | MSS |
|  | **GSE146771** | P0104^†^ | P0104^†^-N | P0104^†^-T | Colon cancer | F | 77 | 4 | 0 | 0 | IIB | MSS |
|  |  | P0305^†^ | P0305^†^-N | P0305^†^-T | Colon cancer | F | 71 | 3 | 1 | 0 | IIA | MSS |
|  |  | P0202 |  | P0202-T | **Rectal cancer** | F | 50 | 2 | 0 | 0 | I | MSS |
|  |  | P0323 |  | P0323-T | Colon cancer | F | 65 | 4 | 1 | 0 | IIIB | MSS |
|  |  | P0408 | P0408-N | P0408-T | Colon cancer | M | 86 | 4a | 0 | 0 | IIB | MSS |
|  |  | P0410 | P0410-N | P0410-T | Colon cancer | F | 66 | 4 | 0 | 0 | IIB | MSS |
|  |  | P0613 | P0613-N | P0613-T | Colon cancer | M | 89 | 4a | 1 | 0 | IIIB | MSS |
|  |  | P1025 | P1025-N | P1025-T | **Rectal cancer** | M | 40 | 4 | 0 | 0 | II | MSS |
|  |  | P1026 | P1026-N | P1026-T | **Rectal cancer** | M | 52 | 4a | 2 | 0 | IIIC | MSS |
|  |  | P0123 | P0123-N | P0123-T | Colon cancer | F | 65 | 4 | 1 | 0 | IIIB | MSI |
